# Presurgical Screening Improves Risk Prediction for Delirium in Elective Surgery of Older Patients: The PAWEL RISK Study

**DOI:** 10.3389/fnagi.2021.679933

**Published:** 2021-07-27

**Authors:** Gerhard W. Eschweiler, Manuel Czornik, Matthias L. Herrmann, Yvonne P. Knauer, Oksana Forkavets, Christine A. F. von Arnim, Michael Denkinger, Olivia Küster, Lars Conzelmann, Brigitte R. Metz, Christoph Maurer, Felix Kentischer, Friederike Deeken, Alba Sánchez, Sören Wagner, Eva Mennig, Christine Thomas, Michael A. Rapp

**Affiliations:** ^1^Geriatric Center, Department of Psychiatry and Psychotherapy, Tübingen University Hospital, Tübingen, Germany; ^2^Department of Neurology and Neurophysiology, Medical Center–University of Freiburg, Freiburg, Germany; ^3^Department of Neurology, University Hospital of Ulm, Ulm, Germany; ^4^Division of Geriatrics, University Medical Center Göttingen, Göttingen, Germany; ^5^Institute for Geriatric Research, Agaplesion Bethesda Ulm and Ulm University, Ulm, Germany; ^6^Helios Clinic for Cardiac Surgery, Karlsruhe, Germany; ^7^Geriatric Center and Department of Geriatric Medicine, ViDia Christian Clinics Karlsruhe, Karlsruhe, Germany; ^8^Department of Social and Preventive Medicine, University of Potsdam, Potsdam, Germany; ^9^Department of Anesthesiology, Klinikum Stuttgart, Stuttgart, Germany; ^10^Department of Geriatric Psychiatry and Psychotherapy, Klinikum Stuttgart, Stuttgart, Germany

**Keywords:** postoperative delirium, elective surgery, cognitive assessment, cognitive impairment, risk prediction, frailty, geriatric assessments, acute encephalopathy

## Abstract

**Introduction:** The number of elective surgeries for patients who are over 70 years of age is continuously growing. At the same time, postoperative delirium (POD) is common in older patients (5–60%) depending on predisposing risk factors, such as multimorbidity, cognitive impairment, neurodegenerative disorders and other dementing disorders, and precipitating factors, such as duration of surgery. Knowledge of individual risk profiles prior to elective surgery may help to identify patients at increased risk for development of POD. In this study, clinical and cognitive risk factors for POD were investigated in patients undergoing various elective cardiac and non-cardiac surgeries.

**Methods:** The PAWEL study is a prospective, interventional trial on delirium prevention. At baseline, 880 inpatients at five surgical centers were recruited for sub-sample PAWEL-R. Multimodal assessments included clinical renal function, medication, American Society of Anesthesiologists (ASA) Physical Status Classification System, geriatric and cognitive assessments, which comprised the Montreal Cognitive Assessment Scale (MoCA), Trail-making Test, and Digit Span backward. Delirium incidence was monitored postoperatively by the Confusion Assessment Method (CAM) and a chart review for up to a week or until discharge. Multivariate regression models and Chi-square Automatic Interaction Detectors (CHAID) analyses were performed using delirium incidence as the primary outcome.

**Results:** Eighteen risk factors were investigated in elective cardiovascular and orthopedic or general surgery. A total of 208 out of 880 patients (24%) developed POD. A global regression model that included all risk variables predicted delirium incidence with high accuracy (AUC = 0.81; 95% CI 0.77, 0.85). A simpler model (clinical and cognitive variables; model CLIN-COG) of 10 factors that only included surgery type, multimorbidity, renal failure, polypharmacy, ASA, cut-to-suture time, and cognition (MoCA, Digit Span backward, and preexisting dementia), however, exhibited similar predictive accuracy (AUC = 0.80; 95% CI 0.76, 0.84).

**Conclusion:** The risk of developing POD can be estimated by preoperative assessments, such as ASA classification, expected cut-to-suture time, and short cognitive screenings. This rather efficient approach predicted POD risk over all types of surgery. Thus, a basic risk assessment including a cognitive screen can help to stratify patients at low, medium, or high POD risk to provide targeted prevention and/or management strategies for patients at risk.

## Introduction

Delirium is characterized by an acute onset and fluctuations in cognition, attention and consciousness (ICD-10, DSM V). The term “delirium” is defined in ICD-10 (F05.0) and ICD-11 (6D70 Delirium Foundation^1^) and characterized by a state of disturbed attention and awareness (i.e., reduced orientation to the environment) that develops over a short period of time and tends to fluctuate (WHO, 2021). In literature, the term “acute encephalopathy” is often used instead of delirium, focusing on pathophysiological aspects of brain dysfunction (Schieveld et al., 2019; Slooter and Stevens, 2020). Delirium is often an incident in medical emergencies (Inouye, 2006), such as sepsis or stroke (Wilson et al., 2020) but also becomes an incident of postoperative delirium (POD), especially in older adults after hip fracture (Kim et al., 2020) or cardiac surgery (Aldecoa et al., 2017). The incidence of POD depends on several predisposing risk factors that are not modifiable, such as age, multimorbidity, stroke, dementing disorders caused by neurodegeneration or other causes, and partially modifiable factors, such as mild cognitive impairment, impaired sensory function, high anxiety levels, and polypharmacy (Guenther et al., 2016) and/or frailty (Leung et al., 2011). Further precipitating factors include the type of surgery, type and duration of anesthesia, and usage of cardiopulmonary bypass (CPB) in heart surgery.

The number of elective surgeries in older adults has dramatically increased within the last years. More than 150,000 knee and hip replacement and 415,000 heart surgery procedures [Operation and Procedure Classification System (OPS) Codes 5–35 to 5–37] have been documented per year in Germany, most of which were performed in patients who are over 70 years of age. On average, hip replacement rates increased by 30% between 2007 and 2017^2^. Especially, older patients with cognitive impairment are overrepresented in German hospitals and are at a high risk for further cognitive decline (Bickel et al., 2018; Nationale Demenz Strategie, 2020, RKI^3^).

A better understanding of the individual risk for the incidence of POD is important, as its occurrence, severity, and duration have been shown to be significantly decreased by modifying risk factors and proper disease management (RR 0.69, 95% CI 0.59–0.81) (Siddiqi et al., 2016; Hughes et al., 2020). Most studies, however, are done in groups with heterogenic surgery procedures without elaborated preoperative cognitive assessments (Aldecoa et al., 2017; Wilson et al., 2020). Preoperative cognitive state is a key risk factor for delirium (Oh et al., 2015) but cannot be estimated objectively in medical or surgical emergencies. Fever, pain, anxiety, blood loss, sedation, and/or lack of time prevent a valid assessment of cognitive domains, such as attention, verbal and non-verbal memory, visuospatial ability, reasoning, and language performance. In contrast to emergency surgery, elective surgery allows preoperative cognitive and further assessments. Objective memory impairments in older adults (Wolfsgruber et al., 2014; Hagen et al., 2015) are not only relevant for the risk of developing dementia but also for developing POD (Aldecoa et al., 2017; Culley et al., 2017; Wilson et al., 2020). Preliminary support is provided for an interaction of diatheses (vulnerabilities) and intra-operative stressors on POD (El-Gabalawy et al., 2017). POD is not only an indicator of brain vulnerability but might also moderate and/or accelerate cognitive decline, especially in patients suffering from dementia (Fong et al., 2015a).

Physical vulnerability (e.g., clinical frailty) is an additional risk factor for POD in cardiac surgery, as recently shown (Itagaki et al., 2020): only older patients who were frail and cognitively impaired showed a significantly increased risk of POD. In elective surgery, most surgeons and anesthesiologists do not assess cognition by screening, although European and American guidelines recommend it at Grade A, which is, however, based on studies with mixed design quality (Aldecoa et al., 2017).

PAWEL-R (“Patientensicherheit, Wirtschaftlichkeit und Lebensqualität” [transl.: “Patient security, economy and life quality”], R for “Risikoschätzung” [transl.: “Risk estimation”]) addresses risk profiles of POD as an add-on study of the PAWEL trial. The PAWEL study focused on the prevention and management of POD (Sanchez et al., 2019) in orthopedic, general, and cardiovascular surgery. The first aim of PAWEL-R was to estimate a more general delirium risk model for elective procedures in cardiac but also for general and orthopedic surgery, such as stepwise cognitive and geriatric assessments. The second aim of PAWEL-R is the development of a pragmatic clinical delirium risk score applicable to patients who are over 70 years of age undergoing elective surgery [PAWEL and the sub-study PAWEL-R are funded by the German Innovation Funds (Innovationsfonds; AZ: VF1_2016-201)].

## Methods

PAWEL-R investigated clinical, psychosocial, and biological risk factors in 880 patients who are over 70 years of age at five surgical centers in southwest Germany: University Hospital Tübingen, Medical Center Stuttgart (*Klinikum Stuttgart*), University Hospital Ulm, University Hospital Freiburg im Breisgau, and the Medical Centers Karlsruhe (*HELIOS Klinik, ViDiaKliniken*). Patients were included if they were scheduled for elective surgery to heart, vessels, abdomen, big joints (hip, knee, shoulder), or spine with an estimated duration of more than 60 min (cut-to-suture time). The estimated clinical survival time was set to be at least 15 months, such that, e.g., surgery of advanced cancer was excluded. Patients who did not speak sufficient German were excluded. The date of first enrollment in the control arm was July 11th, 2017, and the last enrollment was on January 15th, 2019.

### Assessments

The primary endpoint for risk analyses was the incidence of POD assessed by the Confusion Assessment Method (I-CAM) algorithm (Inouye et al., 1990; Thomas et al., 2012) based on DSM V criteria or chart review (Saczynski et al., 2014) within days 1–7 after elective surgery (T2–T9). The prevalence of POD was assessed for 7 days by independent and thoroughly trained raters. The I-CAM algorithm was assessed daily between 1 and 6 p.m. but can be confounded by the fluctuating nature of delirium. Thus, a chart review was added to capture further clinical information, e.g., from night shifts. Chart reviews were performed by medical delirium experts at each center. Further secondary risk factors and blood-based biomarkers were obtained, which are not included in this analysis.

In this analysis, multiple risk factors were addressed, separated into 11 predisposing and four precipitation factors (Aldecoa et al., 2017). The included predisposing factors investigated in the current study were (1) age, (2) multimorbidity (such as Charlson Comorbidity Index, CCI), (3) frailty (Clinical Frailty Scale, CFS; Jones et al., 2005; Dogrul et al., 2020), (4) cognitive impairment such as (a) MoCA Score (Nasreddine et al., 2005), (b) Digit Span backward (Bopp and Verhaeghen, 2005), (c) Trail-Making-Test (TMT), A and B, (5) dementia (present vs. not present), (6) impairment of vision and hearing, (7) alcohol consumption (≥3 drinks/day), (8) smoking (>5 cigarettes/day), (9) polypharmacy, (10) renal failure (creatinine clearance <45 mg/ml), (11) American Society of Anesthesiologists Physical Status Classification System (ASA; Owens et al., 1978).

The four precipitating factors were (1) type of surgery (cardiovascular, orthopedic, and general surgery), (2) cut-to-suture time, (3) type of anesthesia (epidural, larynx mask, intubation), and (4) use of cardiopulmonary bypass (CPB).

### Study Procedure

All the patients were recruited in the surgical departments and surgical outpatient clinics of the five study centers. After checking if they fulfilled inclusion criteria and did not fulfill exclusion criteria, they were asked for written informed consent. In case of moderate dementia or other major cognitive impairment, a legal guardian was asked for written informed consent, and informed assent was obtained by the patient. The patients (and relatives) were interviewed by trained assessors on the day before surgery, or, in case they were invited to preoperative visits, at a maximum distance of 3 weeks prior to surgery. Additional information was extracted from the medical charts. In this study, only patients in the control group and therefore not having received an intervention were analyzed.

For a detailed assessment schedule see the PAWEL trial study protocol (Sanchez et al., 2019). The cognitive assessments (MoCA, TMT, Digit Span backward) were administered on the ward a day before surgery. Regarding the classification of mild cognitive impairment (MCI), we used the original cut-off of <26 points according to Nasreddine et al. (2005) and the recently validated cutoff value of <23 points (Thomann et al., 2020), as the latter showed a high specificity of more than 90% for the detection of MCI in a German-speaking cohort.

The ASA classification (Owens et al., 1978) was assessed by an anesthesiologist at least a day before surgery. The ASA classification is a six-point-scale ranging from ASA score I (a healthy patient), to a maximum ASA score of VI (a brain-dead diagnosed patient, for organ donor purpose). In elective surgery, the ASA score reaches its maximum at score IV [a patient with severe systemic disease that is a constant threat to life, for example recent (<3 months) mitral regurgitation, cerebral vascular accident, transient ischemic attack or coronary artery disease/stents, ongoing cardiac ischemia or severe valve dysfunction, severe reduction of ejection fraction, shock, sepsis, and others^4^]. More details of ASA classification can be found in Supplement 2-Table 1.

### Statistics

Assessments and data from patients without POD were compared with those from patients who suffered from POD using *t-*tests or chi-squared-tests. For each assessment and data evaluation, costs and duration were estimated to calculate the effort (time × costs). Assessments and data that were only available for <90% of the patients were not included in the final analyses.

A logistic regression model was used to develop the prediction score by assessing the association between the potential risk factors and the presence or absence of POD. Selection and weighting of the predictors of the final model took place through a manual forward selection of the candidate predictors based on the odds ratio (OR). To calculate an area under the curve (AUC), we applied receiver operating characteristic (ROC) curves to the data. The optimal cutoff point to discriminate between the low and high probability of delirium was chosen by means of the Youden index.

To identify symptom constellations reflecting delirium risk, we computed decision trees as a type of cluster analysis. The decision trees were created with the chi-squared automatic interaction detection (CHAID) growing method using the presence of delirium as the dependent variable, and potential risk factors as independent variables. Hierarchical cluster analysis was performed to account for the relationship between predisposing factors. The level of significance was set at α = 0.05 (two-tailed, Bonferroni-corrected). The chi-squared-tests in the analysis were Bonferroni-corrected for multiple testing with a minimal parent size of 80 subjects and a child size of 40.

Statistical analyses were performed using SPSS 26.0 (IBM SPSS Inc., Chicago, IL, United States). DeLong's tests for two ROC curves in a correlated population were applied to compare the AUCs of all models undergoing multivariate regression (DeLong et al., 1988) using the open source program R^5^.

## Results

### Study Population Characteristics

**Figure 1:**
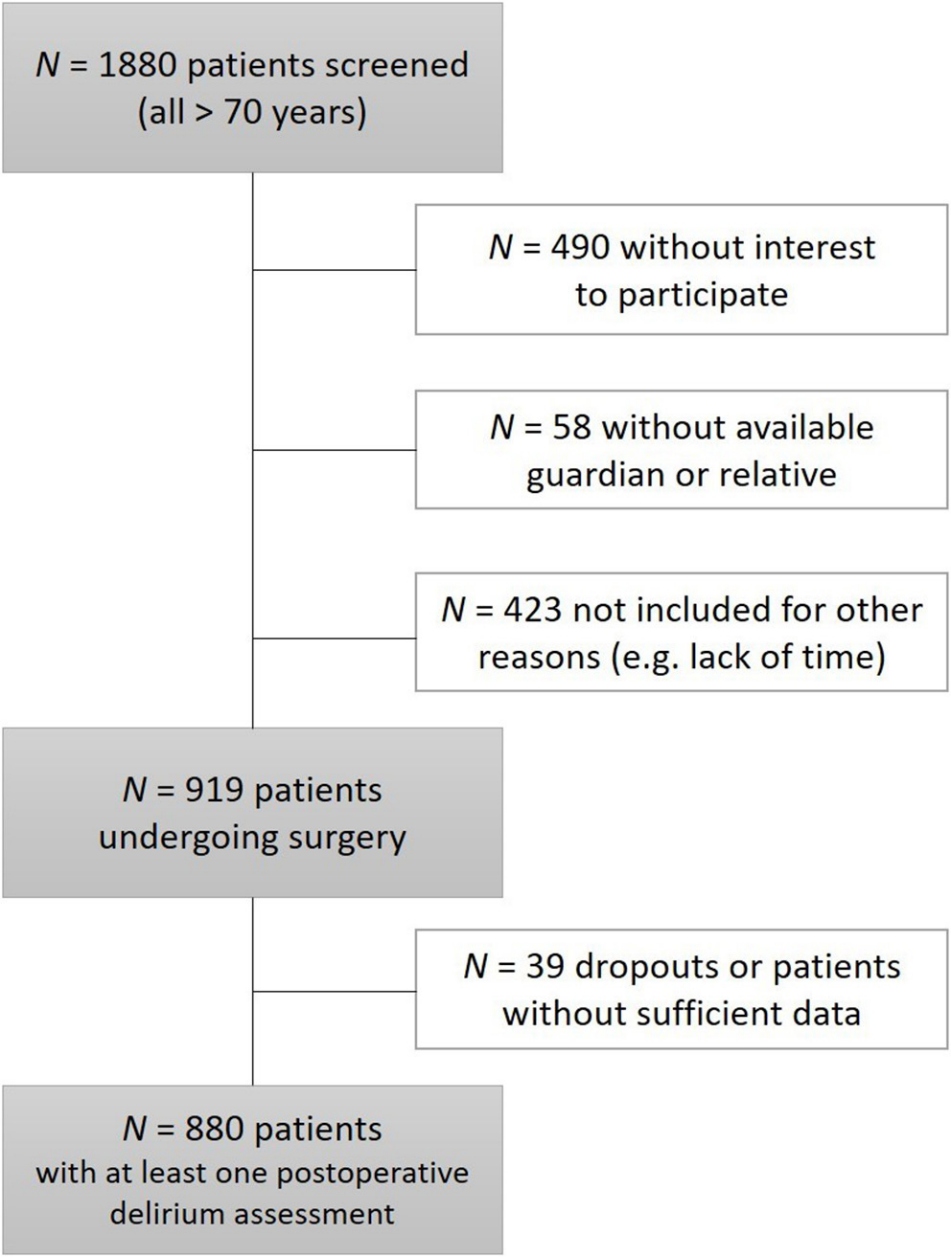
Overview of enrollment and exclusion of patients in the study.

A total of 1,880 patients older than 70 years fulfilled the inclusion criteria before or at admission to hospital and were screened in one of the five centers. Four hundred ninety patients had no interest in participating in the study, and 423 were not included because of lack of time, administrative scheduling, or rescheduling of surgeries. Fifty-eight patients were excluded because of lack of consent from relatives or legal guardians. Of the remaining participants, 919 received elective surgery. Thirty-nine patients dropped out after index surgery, of whom nine received a second surgery within the first week. Thus, perioperative data from 880 patients with at least a postoperative delirium assessment were available for data analysis. Figure 1 illustrates the enrollment and exclusion of patients leading to the sample analyzed as follows.

Of these 880 patients, 329 underwent cardiovascular and 551 orthopedic-general surgeries, and 397 received surgery to the joints (hip, knee, or shoulder). Eighty-three patients received abdominal surgery, 48 received surgery of the spine, and 23 received other surgical procedures. Cardiovascular surgery included 280 cardiac (bypass, valve, or mixed surgery) and 49 vascular surgeries. Basic preoperative characteristics for the whole sample are listed in Table 1.

**Table 1:** Overview of basic characteristics for whole sample.

**Variable**	**Is measured in**	**Mean (+/- SD)**	**Range**	***N*** **(available data)**	**Percentage available data (%)**	**Defined categories**	**N (in each category)**	**Type of assessment**	**Duration of assessment (in minutes)**	**Effort**
Age	Years	77.82 (± 4.87)	70.07–96.17	880	100	70–75 years	272	B	0	1
						75–80 years	332			
						80–85 years	188			
						>85 years	76			
Gender	Categorical: Female/Male			880	100	Female	434	B	0	1
						Male	446			
Education	Years	12.23 (± 2.98)	3–18	863	98.07			A	1	1
ASA	Score	2.81 (± 0.62)	I–IV	871	98.98	Score I + II	240	A	3	1
						Score III	545			
						Score IV	86			
Clinical frailty score	Score	3.59 (± 1.35)	1–8	871	98.98	Score 1–3	476	A, pm	1	1
						Score 4–8	395			
Polypharmacy	Drugs per day			880	100	≤5 drugs	377	A, pm	2	1
						>5 drugs	503			
						≤10 drugs	771			
						>10 drugs	109			
						≤5 drugs	377			
						6–10 drugs	394			
						>10 drugs	109			
Multimorbidity	Number of diseases	6.19 (± 2.57)	0–20	880	100	≤4 diseases	236	A		2
						5–8 diseases	563			
						≥9 diseases	81			
Charlson comorbidity index	Score	2.16 (± 0.92)	1–4	880	100	Score 1 + 2	605	A	2	2
						Score 3 + 4	275			
MoCA (nasreddine)	Points	26.85 (± 4.20)	5–29	855	97.16	≥26 points	276	C, V, A	13	3
						<26 points	580			
MoCA (<23)						≥23 points	565			
						<23 points	291			
TMT A	Seconds	48.71 (± 28.86)	20–180	777	88.30			C, V	4	2
	z-Score	0.17 (± 1.77)	−9.75–3.36			≥-1.28 z	699			
						<-1.28 z	65			
TMT B	Seconds	159.71 (± 70.62)	22–300	732	83.41			C, V	6	2
	z-Score	−0.59 (± 1.50)	−6.16–3.16			≥-1.28 z	534			
						<-1.28 z	187			
Digit span	Longest Range	5.11 (± 2.12)	0–14	819	93.07	≥5 points	488	C, V	4	2
						<5 points	331			
Pre-existing dementia	Categorical: Yes/No			880	100	No	868	A	3	1
						Yes	14			
Alcohol consumption	Categorical:			877	99.66	≤3 drinks	874	A, pm	3	1
	Drinks per day					>3 drinks	3			
	Categorical:					≤3 drinks	852			
	Drinks per week					>3 drinks	25			
Smoking	Categorical:			880	100	≤5 cigarettes	840	A, pm	1	1
	Cigarettes per day					>5 cigarettes	40			
Auditory impairment	Categorical:			865	98.30	No ear	487	S, pm	2	1
	Ears impaired					One ear	182			
						Both ears	196			
Visual impairment	Categorical:			839	95.34	No	658	S, pm	2	1
	Vision impaired					Yes	181			
Sensory impairment	Categorical:			835	94.89	None	86	S, pm	4	2
	Number of senses impaired					One sense	478			
						Both senses	271			
Hyposmia	Number of correctly identified Sniffin-Sticks	9.03 (± 2.31)	0–12	728	82.73	No Yes	499 229	S	10	2
Cut-to-suture-time	Minutes	146.84 (± 84.36)	28–543	879	99.89	<90 min.	305	P		
						90–180 min.	296			
						>180 min.	278			
Renal failure	Creatinine-Clearance in mmol/l	193.95 (± 1375.68)	0.4–9990	880	100	>45 mmol/ml <45 mmol/ml	752 108	L, pm	1	2
Cardio-pulmonary bypass	Categorical: Yes/No			876	99.55	No Yes	638 238	P		
Surgery type	Categorical:			880	100	No	551	P		
	Cardio-vascular Surgery					Yes	329			

*Overview of basic characteristics for all the patients (n = 880). All the patients underwent elective surgery and were assessed for having delirium at least once. For continuous data, mean, standard deviation, and range are given. If categories were defined or only categorical data were assessed, the categories defined and the number of patients in each group is given. Additionally, the type of assessment is defined (B, basic data; A, anamnestic or personal history; C, cognitive assessment; S, sensory assessment; P, perioperative assessment; pm, indicates if variable is partially modifiable). Duration of assessment in minutes as well as the overall effort on a scale from 1 to 3 is stated as well*.

The final sample included 49% female patients, with a mean age of 77.8 years (±4.9 years). The participants reported a mean of 12.2 years of education (±3 years; including professional or academic training). Mean scores for all assessments are given in Table 1. In addition, the time needed for each assessment execution or gathering of data as well as the estimated relative effort (time × costs) is given.

Multimorbidity was assessed by history-taking and was estimated at a mean of 6.2 disorders (±2.6 disorders) on average. The Charlson Comorbidity Index (CCI) is a weighted clinical score of 19 disorders (Charlson et al., 1987). The mean of the CCI as a measure of multimorbidity was 2.16 (±.92) with a median of 2 in the total sample. The different morbidity groups classes exhibited 25.3% of patients in class 1 (lowest morbidity), 43.5% in class 2, 21% in class 3, and 10.2% in class 4 (highest morbidity). Polypharmacy, defined as 10 drugs or more, was found in 12% of the patients.

As for perioperative factors, the most frequent type of anesthesia was general anesthesia with intubation (75.1%), followed by epidural spinal anesthesia (15.8%) and larynx mask anesthesia (7.6%). The mean cut-to-suture time was 148 min (±85 min). Only 17% of the orthopedic and general surgeries took longer than 180 min, while 67% of cardiac surgical procedures took longer than 180 min. In the cardiovascular surgery group, 87% of the patients received a CPB.

Sixty-seven percent of the patients suffered from cognitive impairment as defined by a cut-off of <26 points in the MoCA score (Nasreddine et al., 2005). Implementation of an adapted MoCA cut-off of <23 points (Thomann et al., 2020) identified 34% of the patients as cognitive impaired before surgery. Data availability was higher than 90% for most assessments, but was below 90% for TMT-A and TMT-B, because of visual, psychomotor or cognitive impairments, exhaustion, or time restrictions. Among the patients, 1.5% (*n* = 14) had received a dementia diagnosis at the time of enrollment. Visual or auditory impairments were present in more than 20%, and 2.8% (*n* = 25) reported to consume three or more drinks per week and 4.5% (*n* = 40) were active smokers.

### Preoperative Differences Between Patients With vs. Without POD

Among the patients, 208 (24%) suffered from POD at least on 1 day during the first postoperative week (from T2 to T9). Delirium prevalence was 36% in the cardiovascular group and 16% in the orthopedic-general surgery group. We, thus, stratified the data analytic approach to analyze both major surgery groups together in a first step.

Comparisons between the groups of patients with and without POD regarding preoperative and perioperative risk factors are given in Table 2. While age did not significantly differ between the groups [*t*_(878)_ = −1.8, *p* = 0.073], multimorbidity was higher [*t*_(878)_ = −5.08, *p* < 0.001], cut-to-suture time was nearly 1 h longer [*t*_(278.69)_ = −7.85, *p* < 0.001], and the preoperative cognitive state represented by the MoCA score [*t*_(253.46)_ = 5.27, *p* < 0.001] as well as by the Digit Span backward [*t*_(817)_ = 3.31, *p* < 0.001] was lower in patients suffering from POD than in patients without POD. An ASA score of III or IV was present in 67% of the patients without POD and in 91% of the patients with POD and, therefore, higher in the latter group [*t*_(869)_ = −9.75, *p* < 0.001].

**Table 2:** Overview and comparison of basic characteristics as a function of POD.

	**Study subjects without delirium (** ***n*** **= 672)**					**Study subjects with delirium (** ***n*** **= 208)**							
**Variable**	**Mean (± SD)**	***N*** **(available data)**	**Percentage available data (%)**	**Categories**	***N***	**Mean (± SD)**	***N*** **(available data)**	**Percentage available data (%)**	**Categories**	***N***	***t***	**χ^2^**	***p***
Age	77.67 (± 4.75)	672	100	70–75 years	214	78.36 (± 5.22)	208	100	70–75 years	58	−1.80		0.073
				75–80 years	253				75–80 years	79			
				80–85 years	142				80–85 years	46		2.72	0.437
				>85 years	53				>85 years	23			
Gender		672	100	Female	344		208	100	Female	90		3.68	0.055^(*)^
				Male	328				Male	118			
Education	12.25 (± 3.02)	661	98.36			12.25 (± 2.85)	202	97.12			0.42		0.674
ASA	**2.70 (± 0.59)**	665	98.96	Score I + II	**221**	**3.16 (± 0.58)**	206	99.04	Score I + II	**19**	–**9.75**		<0.001^*^
				Score III	**410**				Score III	**135**		**97.83**	<0.001^*^
				Score IV	**34**				Score IV	**52**			
Clinical frailty scale	**3.50 (± 1.30)**	665	98.96	Score 1–3 Score 4–8	**380** **285**	**3.89 (± 1.48)**	206	99.04	Score 1–3 Score 4–8	**96** **110**	–**3.42**	**6.63**	<0.001^*^ 0.010^*^
Polypharmacy		672	100	≤5 drugs	**311**		208	100	≤5 drugs	**66**		**13.14**	<0.001^*^
				>5 drugs	**361**				> 5 drugs	**142**			
				≤10 drugs	**602**				≤10 drugs	**169**		**9.41**	0.002^*^
				>10 drugs	**70**				>10 drugs	**39**			
				≤5 drugs	**311**				≤5 drugs	**66**		**18.12**	<0.001^*^
				6–10 drugs	**291**				6–10 drugs	**103**			
				>10 drugs	**70**				>10 drugs	**39**			
Multimorbidity	**5.95 (± 2.49)**	672	100	≤4 diseases	**205**	**6.97 (± 2.68)**	208	100	≤4 diseases	**31**	–**5.08**		<0.001^*^
				5–8 diseases	**418**				5–8 diseases	**145**		**27.12**	<0.001^*^
				≥9 diseases	**49**				≥9 diseases	**32**			
Charlson comorbidity index	**2.11 (± 0.91)**	672	100	Score 1 + 2 Score 3 + 4	473 199	**2.32 (± 0.92**	208	100	Score 1 + 2 Score 3 + 4	133 75	–**2.92**	2.78	0.004^*^ 0.095^(*)^
MoCA	**27.34 (± 3.66)**	657	98.06	≥26 points	**233**	**25.20 (± 5.34)**	197	94.71	≥26 points	**43**	**5.27**		<0.001^*^
				<26 points	**425**				<26 points	**154**		**12.18**	<0.001^*^
				≥23 points	**462**				≥23 points	**103**		**20.95**	<0.001^*^
				<23 points	**196**				<23 points	**84**			
TMT A	**46.00 (± 24.86)**	600	89.29			**56.58 (± 38.51)**	177	85.10			–**3.45**		<0.001^*^
	**0.30 (± 1.59)**			≥-1.28 z	**553**	**−0.26 (± 2.22)**			≥-1.28 z	**146**	**3.10**		0.002^*^
				< -1.28 z	**38**				<-1.28 z	**27**		**13.32**	<0.001^*^
TMT B	**152.80 (± 68.11)**	570	84.82			**184.02 (± 74.05)**	162	77.88			–**4.82**		<0.001^*^
	**-0.46 (± 1.44)**			≥-1.28 z	**431**	–**1.02 (± 1.61)**			≥-1.28 z	**129**	**4.19**		<0.001^*^
				<-1.28 z	**130**				<-1.28 z	**59**		**9.71**	0.002^*^
Digit span	**5.25 (± 2.08)**	624	92.86	≥5 points	**397**	**4.65 (± 2.21)**	195	93.75	≥5 points	**104**	**3.51**		<0.001^*^
				<5 points	**227**				<5 points	**91**		**17.4**	<0.001*
Pre-existing dementia		672	100	No Yes	**671**		208	100	No Yes	**195** **13**		**33.97**	<0.001^*^
Alcohol consumption		670	99.70	≤3 drinks/day	668		207	99.52	≤3 drinks/day	206		<0.001	1.00
				>3 drinks/day	2				>3 drinks/day	1			
				≤3 drinks/week	651				≤3 drinks/week	201		<0.001	1.00
				>3 drinks/week	19				>3 drinks/week	6			
Smoking		672	100	≤5 cigarettes	644		208	100	≤5 cigarettes	196		0.61	0.436
				>5 cigarettes	28				>5 cigarettes	12			
Auditory impairment		661	98.36	No ear	381		204	98.08	No ear	106		3.07	0.215
				One ear	139				One ear	43			
				Both ears	141				Both ears	55			
Visual impairment		637	94.79	No	504		202	97.12	No	154		0.59	0.441
				Yes	133				Yes	48			
Sensory impairment		635	94.49	None	62		200	96.15	None	24		3.58	0.167
				One sense	375				One sense	103			
				Both senses	198				Both senses	73			
Hyposmia	**9.20 (± 2.15)**	560	83.43	No	**397**	**8.46 (± 2.69)**	168	80.77	No	**102**	**3.26**		0.001^*^
				Yes	**163**				Yes	**66**		**5.75**	0.017^*^
Cut-to-suture-time	**132.99 (± 73.74)**	672	100	<90 min	**256**	**191.82 (± 99.81)**	207	99.52	<90 min	**49**	–**7.85**		<0.001^*^
				90–180 min	**252**				90–180 min	**44**		**68.95**	<0.001^*^
				>180 min	**164**				>180 min	**114**			
Renal failure	223.94 (± 1478.90)	672	100	>45 mmol/ml	**585**	97.09 (± 977.13)	208	100	>45 mmol/ml	**167**	1.16		0.245
				<45 mmol/ml	**71**				<45 mmol/ml	**37**		**6.93**	0.008^*^
Cardio-pulmonary bypass		668	99.40	No	**526**		208	100	No	**113**		**46.68**	<0.001^*^
				Yes	**142**				Yes	**95**			
Surgery type		672	100	No	**463**		208	100	No	**88**		**46.85**	<0.001^*^
				Yes	**209**				Yes	**120**			

Overview of basic characteristics and pre- as well as perioperative assessed data of the patients without delirium (n = 672) compared with data of the patients with delirium (n = 208). For continuous data, mean, standard deviation, and range are given. If categories were defined or only categorical data were assessed, the categories defined and the number of patients in each group is given. Test statistics comparing these groups for differences on all available data are stated as well (t-tests were conducted on continuous data, χ^2^-tests on categorical; if both types of data were present, both tests were conducted). Statistically significant tests results are highlighted in bold type, significant p-values are marked with *, and p-values at a significance level of .05 to 0.1 are marked with (*).

The cognitive subscores of the MoCA are shown in Supplement 2-Table 2. In the non-parametric tests, all subscores beside naming showed significant differences between non-delirious and delirious patients, but differences in the non-parametric group tests were most pronounced for the memory domain, followed by orientation and then language.

Preoperative and perioperative risk factors were analyzed for patients undergoing only cardiovascular surgery and for patients undergoing only orthopedic or general surgery analogy. Test statistics comparing patients with and without POD within these subsamples for differences between risk factors can be found in the Supplementary Material.

### Regression Analyses: Model FULL

In the first step, 18 potential risk factors were analyzed in univariate logistic regressions for the total group of 880 patients by Bonferroni adjustment throughout. Results from the univariate analysis can be found in Table 3. Subsequently, a multivariate logistic analysis was conducted by which significant predictors from the univariate regression analyses were analyzed in the model FULL for all the 880 patients (see Table 3). In the univariate analyses, all ORs were significant, except for age group, alcohol consumption and smoking, as well as auditory and visual impairment, probably reflecting selection biases in elective surgery. The CCI showed marginal predictive power. In the multivariate analysis, ORs were significantly increased for the following five factors: ASA, multimorbidity, cut-to-suture time, clinical frailty scale and cognition, either dementia or MCI (defined as MoCA score <23). The overall explained variance of these 18 risk factors calculated using Nagelkerke's Pseudo-*R*^2^ was 33.4% (*F* = 21.56, *p* < 0.001) and an AUC of 0.81 (95% CI 0.77, 0.85) was computed. As dementia was only reported in 14 patients, of whom 13 developed POD, a second multivariate logistic regression was calculated without dementia as a potential risk factor. In this analysis, the impact of cognitive impairment defined by MoCA <23 increased from a marginally significant OR of 1.59 (95% CI 0.98, 2.58; *p* = 0.054) in the primary multivariate regression analysis with dementia included to a significant predictive OR of 1.8 (95% CI 1.12, 2.89; *p* = 0.015). Results of this adapted regression analysis can be found in the Supplementary Material.

**Table 3:** Univariate and multivariate logistic regression analysis (FULL model).

			**Univariate regression analysis**			**Multivariate Regression Analysis: Model FULL**		
	**Variable**	**Categories**	***N*** **(with delirium); Percentage**	**OR [95%-CI]**	***P***	**N (with delirium); Percentage**	**OR [95%-CI]**	***p***
	Age	70–75 years	272 (58); 21.32%			248 (53); 21.37%		
		75–80 years	332 (79); 23.80%	1.15 [0.79–1.70]	0.471	291 (70); 24.06%	1.05 [0.65–1.72]	0.837
		80–85 years	188 (46); 24.47%	1.20 [0.77–1.86]	0.428	157 (38); 24.20%	1.12 [0.61–2.03]	0.719
		>85 years	76 (23); 30.26%	1.60 [0.90–2.81]	0.105	54 (16); 29.63%	1.56 [0.66–3.53]	0.297
	ASA	Score I + II	240 (19); 7.92%			207 (16); 7.73%		
		Score III	545 (135); 24.77%	**3.83 [2.36–6.55]**	<0.001^*^	464 (113); 24.35%	**2.21 [1.16–4.40]**	0.019^*^
		Score IV	86 (52); 60.47%	**17.79 [9.57–34.40]**	<0.001^*^	79 (48); 60.76%	**6.67 [2.88–15.92]**	<0.001^*^
	Clinical frailty score	Score 1–3	476 (96); 20.17%			432 (86); 19.91%		
		Score 4–8	395 (110); 27.85%	**1.53 [1.12–2.09]**	0.008^*^	318 (91); 28.62%	1.79 [1.14–2.85]	0.012^*^
Polypharmacy	Polypharmacy (≤5 drugs)	≤5 drugs	377 (66); 17.51%			330 (57); 17.27%		
		>5 drugs	503 (142); 28.23%	**1.85 [1.34–2.59]**	<0.001^*^	420 (120); 28.57%	1.01 [0.64–1.62]	0.951
	Polypharmacy (≤10 drugs)	≤10 drugs	771 (169); 21.92%			660 (145); 21.97%		
		>10 drugs	109 (39); 35.78%	**1.98 [1.29–3.03]**	0.002^*^	90 (32); 35.56%	1.28 [0.68–2.36]	0.438
	Polypharmacy (≤5 drugs, <10 drugs)	≤5 drugs	377 (66); 17.51%					
		6–10 drugs	394 (103); 26.14%	**1.67 [1.18–2.37]**	0.004^*^			
		>10 drugs	109 (39); 35.78%	**2.63 [1.63–4.21]**	<0.001^*^			
	Multimorbidity	≤4 diseases	236 (31); 13.14%			204 (26); 12.75%		
		5–8 diseases	563 (145); 25.75%	**2.29 [1.52–3.55]**	<0.001^*^	473 (122); 25.79%	**1.91 [1.12–3.33]**	0.020^*^
		≥9 diseases	81 (32); 39.51%	**4.32 [2.41–7.78]**	<0.001^*^	72 (29); 40.28%	**3.89 [1.68–9.02]**	0.001^*^
	Charlson comorbidity index	Score 1 + 2	604 (133); 22.02%			515 (114); 21.71%		
		Score 3 + 4	274 (75); 27.37%	1.34 [0.96–1.88]	0.080^(*)^	235 (63); 26.81%	0.63 [0.38–1.03]	0.067^(*)^
Pre-existing cognitive impairment	MoCA (nasreddine)	≥26 points	276 (43); 15.58%			244 (42); 17.21%		
		<26 points	579 (154); 26.60%	**1.96 [1.36–2.88]**	<0.001^*^	506 (135); 26.68%	1.17 [0.70–1.99]	0.547
	MoCA (<23)	≥23 points	565 (103); 18.23%			509 (98); 19.25%		
		<23 points	290 (94); 32.41%	**2.15 [1.55–2.98]**	<0.001^*^	241 (79); 32.78%	1.61 [0.99–2.61]	0.054^(*)^
	TMT A	≥-1.28 z	699 (146); 20.89%					
		<-1.28 z	65 (27); 41.54%	**2.69 [1.58–4.54]**	<0.001^*^			
	TMT B	≥-1.28 z	533 (102); 19.14%					
		<-1.28 z	187 (57); 30.48%	**1.85 [1.26–2.70]**	0.001^*^			
	Digit Span	≥5 points	488 (91); 18.65%			455 (85); 18.68%		
		<5 points	331 (104); 31.42%	**2.00 [1.44–2.77]**	<0.001^*^	295 (92); 31.19%	1.46 [0.96–2.22]	0.079^(*)^
	Pre-existing dementia	No	866 (195); 22.52%			740 (168); 22.70%		
		Yes	14 (13); 92.86%	**44.73 [8.83–815.22]**	<0.001^*^	10 (9); 90.00%	**29.60 [4.99–567.88]**	0.002^*^
Alcohol consumption	Alcohol consumption per day	≤3 drinks	874 (206); 23.57%					
		>3 drinks	3 (1); 33.33%	1.62 [0.08–17.01]	0.694			
	Alcohol consumption per week	≤3 drinks	852 (201); 23.59%					
		>3 drinks	25 (6); 24.00%	1.02 [0.37–2.46]	0.962			
	Smoking on a day	≤5 cigarettes	840 (196); 23.33%					
		>5 cigarettes	40 (12); 30.00%	1.41 [0.68–2.76]	0.334			
Significant sensory impairment	Auditory impairment	No ear	487 (106); 21.77%			433 (92); 21.25%		
		One ear	182 (43); 23.63%	1.11 [0.74–1.66]	0.607	157 (38); 24.20%	1.37 [0.81–2.31]	0.234
		Both ears	196 (55); 28.06%	1.41 [0.96–2.04]	0.080^(*)^	160 (47); 29.38%	1.35 [0.80–2.26]	0.250
	Visual impairment	No	658 (154); 23.40%					
		Yes	181 (48); 26.52%	1.18 [0.81–1.71]	0.386			
	Sensory impairment	None	86 (24); 27.91%					
		One sense	477 (103); 21.59%	0.71 [0.43–1.21]	0.195			
		Both senses	270 (73); 27.04%	0.95 [0.56–1.66]	0.860			
	Hyposmia	No	499 (102); 20.44%					
		Yes	229 (66); 28.82%	**1.58 [1.10–2.25]**	0.013^*^			
	Cut-to-suture-time	<90 min	305 (49); 16.07%			252 (40);15.87%		
		90–180 min	296 (44); 14.86%	0.91 [0.58–1.42]	0.684	248 (36); 14.52%	0.57 [0.31–1.05]	0.072^(*)^
		>180 min	278 (114); 41.01%	**3.63 [2.48–5.39]**	<0.001^*^	250 (101); 40.40%	**1.93 [1.00–3.71]**	0.047^*^
	Renal failure	>45 mmol/ml	752 (167); 22.21%			657 (147); 22.37%		
		<45 mmol/ml	108 (37); 34.26%	**1.83 [1.17–2.80]**	0.006^*^	93 (30); 32.26%	1.32 [0.72–2.40]	0.365
	Cardio-pulmonary bypass	No	639 (113); 17.68%			524 (87); 16.60%		
		Yes	237 (95); 40.08%	3.11 [2.24–4.33]	<0.001^*^	226 (90); 39.82%	1.75 [0.89–3.52]	0.108
	Surgery type	No	551 (88); 15.97%			446 (68); 15.25%		
		Yes	329 (120); 36.47%	3.02 [2.20–4.17]	<0.001^*^	304 (109); 35.86%	1.36 [0.66–2.74]	0.392

Results from univariate and multivariate logistic regression analysis in order to identify predictors for delirium risk. All the data were grouped into the categories stated. Only variables significant in the univariate analysis and with more than 90% of data available (see Table 2) were included in the multivariate analysis (n = 750 patients), of which n = 177 (23.6%) suffered from delirium. Pseudo-R^2^ for the multivariate regression model was 0.333, an AUC of 0.81 [95% CI (0.773–0.848)] was calculated; the overall F-test reached significance (F = 20.28, p < 0.001). After the maximal Youden-index method, sensitivity was calculated with 0.689 and specificity with 0.818. A positive predictive value (PPV) of 0.54 and a negative predictive value (NPV) of 0.895 was computed for this FULL model. Statistically significant tests results are highlighted in bold type, significant p-values are marked with *, and p-values at a significance level of .05 to 0.1 are marked with (*).

### Stepped Assessment of Less Complex Models

**Figure 2:**
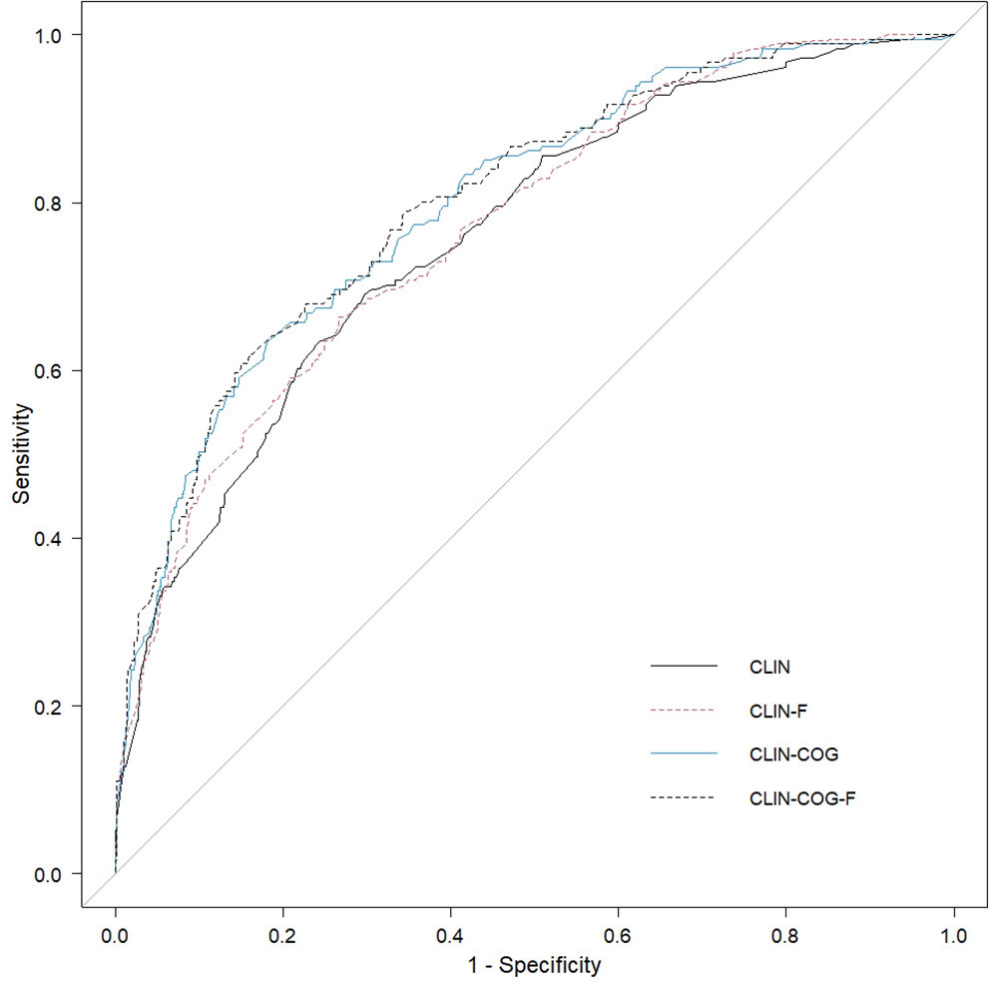
Comparison of ROC curves constructed for all models. The clinical model (CLIN) included the seven clinical variables, model CLIN-F encompasses the dichotomized frailty index, and models CLIN-COG and CLIN-COG-F include cognition as well.

For reasons of efficiency, less complex models for potential usage in hospitals were computed for *n* = 770 patients, for whom all data were available. Risk factors were included as they reached significance in the upper model FULL and if more than 90% of data were available in the whole sample. Table 4 includes the results of the multivariate regression analyses for all of these four models: All clinical and perioperative variables are included in model CLIN, model CLIN-F encompasses the dichotomized frailty index, and models CLIN-COG and CLIN-COG-F include cognition as well. Table 5 shows values and test statistics of likelihood comparison tests as well as AUCs and corresponding DeLong's test statistics for the comparison of two ROC curves. Figure 2 illustrates the ROC curves constructed for all models.

**Table 4:** Multivariate logistic regression analysis to identify the less complex models.

				**Model CLIN**		**Model CLIN-F**		**Model CLIN-COG**		**Model CLIN-COG-F**	
Basic information	*N* (*N* with delirium)			771 (181); 23.48%		771 (181); 23.48		771 (181); 23.48		771 (181); 23.48%	
	Nagelkerke's pseudo-*R*^2^			0.242		0.256		0.304		0.313	
	*F*			30.41		29.09		30.14		28.78	
	*p*			<0.001*		<0.001*		<0.001*		<.001*	
	AUC [95%-CI]			0.760 [0.721–0.800]		0.769 [0.731–0.808]		0.798 [0.762–0.835]		0.801 [0.765–0.838]	
	**Variable**	**Categories**	***N*** **(** ***N*** **with delirium); Percentage**	**OR [95%-CI]**	***p***	**OR [95%-CI]**	***P***	**OR [95%-CI]**	***p***	**OR [95%-CI]**	***P***
Clinical	ASA	Score I + II	215 (17); 7.91%								
		Score III	477 (116); 24.32%	**2.25 [1.24–4.24]**	0.009*	**2.09 [1.15–3.96]**	0.019*	**2.22 [1.20–4.28]**	0.013*	**2.08 [1.12–4.02]**	0.024*
		Score IV	79 (48); 60.76%	**7.75 [3.55–17.42]**	<0.001*	**7.02 [3.19–15.90]**	<0.001*	**6.98 [3.12–16.09]**	<0.001*	**6.44 [2.86–14.93]**	<0.001*
	Multimorbidity	≤4 diseases	210 (26); 12.38%								
		5–8 diseases	489 (126); 25.77%	**1.85 [1.13–3.09]**	0.016*	**1.83 [1.13–3.06]**	0.017*	**1.79 [1.08–3.04]**	0.027*	**1.77 [1.07–3.01]**	0.029*
		≥9 diseases	72 (29); 40.28%	**3.61 [1.81–7.26]**	<0.001*	**3.27 [1.62–6.64]**	<0.001*	**3.36 [1.63–6.94]**	0.001*	**3.07 [1.48–6.40]**	0.003*
	Cut-to-suture time	<90 min	260 (41); 15.77%								
		90–180 min	256 (38); 14.84%	0.67 [0.38–1.15]	0.145	0.62 [0.35–1.07]	0.088^(*)^	0.63 [0.35–1.11]	0.114	0.59 [0.33–1.05]	0.077^(*)^
		>180 min	255 (102); 40.00%	1.69 [0.93–3.03]	0.082^(*)^	1.62 [0.89–2.93]	0.112	1.69 [0.92–3.10]	0.093^(*)^	1.63 [0.88–3.01]	0.120
	Renal failure	>45 mmol/ml	672 (148); 22.02%								
		<45 mmol/ml	99 (33); 33.33%	1.58 [0.94–2.61]	0.080^(*)^	1.46 [0.86–2.44]	0.155	1.49 [0.87–2.53]	0.139	1.40 [0.81–2.38]	0.227
	Polypharmacy	≤5 drugs	339 (60); 17.70%								
		6–10 drugs	338 (88); 26.04%	1.18 [0.77–1.81]	0.447	1.08 [0.70–1.66]	0.743	1.03 [0.66–1.61]	0.886	0.96 [0.61–1.51]	0.866
		> 10 drugs	94 (33); 35.11%	1.66 [0.90–3.05]	0.101	1.34 [0.71–2.51]	0.358	1.37 [0.72–2.60]	0.332	1.16 [0.59–2.22]	0.666
	Cardio-pulmonary Bypass	No	544 (90); 16.54%								
		Yes	227 (91); 40.09%	1.45 [0.77–2.80]	0.252	1.59 [0.84–3.10]	0.159	1.54 [0.81–3.00]	0.191	1.66 [0.87–3.26]	0.129
	Surgery type	Ortho./Visc.	464 (70); 15.09%								
		Cardio-vasc.	307 (111); 36.16%	1.09 [0.56–2.07]	0.804	1.26 [0.64–2.43]	0.503	1.32 [0.67–2.54]	0.420	1.49 [0.74–2.91]	0.253
Cognition	MoCA	≥23 points	517 (100); 19.34%								
		<23 points	254 (81); 31.89%					**1.95 [1.28–2.98]**	0.002*	**1.82 [1.19–2.80]**	0.006*
	Digit span	≥5 points	460 (86); 18.70%								
		<5 points	311 (95); 30.55%					**1.50 [1.01–2.25]**	0.047*	1.49 [0.99–2.24]	0.053^(*)^
	Pre-existing dementia	No	761 (172); 22.60%								
		Yes	10 (9); 90.00%					**26.17 [4.49–498.64]**	0.003*	**28.35 [4.82–541.97]**	0.002*
Frailty	Clinical frailty score	Score 1–3	441 (89); 20.18%								
		Score 4–8	330 (92); 27.89%			**1.88 [1.23–2.90]**	0.004*			**1.74 [1.12–2.72]**	0.015*

Results from multivariate logistic regression analysis comparing four possible models with higher economical practicability. All the data were grouped into the categories stated. To ensure the possibility of statistical comparison of all models, only the patients were included, for whom data in all the models were available. Statistically significant tests results are highlighted in bold type, significant p-values are marked with *, and p-values at a significance level of .05 to 0.1 are marked with (*).

**Table 5:** Comparison of the four models.

		**Pseudo–*R*^2^**	**Model CLIN**	**Model CLIN-F**	**Model CLIN-COG**	**Model CLIN-COG-F**
Likelihood ratio tests	Model CLIN	0.242		***χ*** **^2^_(1)_ = 8.45,** ***p*** = 0.004*	***χ*** **^2^_(3)_ = 38.81,** ***p*** < 0.001*	***χ*** **^2^_(4)_ = 44.80,** ***p*** < 0.001*
	Model CLIN-F	0.256			***χ*** **^2^_(2)_ = 30.37,** ***p*** < 0.001*	***χ*** **^2^_(3)_ = 36.36,** ***p*** < 0.001*
	Model CLIN-COG	0.304				***χ*** **^2^_(1)_ = 5.99,** ***p*** = 0.014*
	Model CLIN-COG-F	0.313				
		**AUC [95%-CI]**				
DeLong's tests for two ROC-curves in a correlated population	Model CLIN	0.760 [0.721–0.800]		*z* = −1.41, *p* = 0.159	***z*****= −3.61**, ***p***< 0.001*	***z*****= −3.50**, ***p***< 0.001*
	Model CLIN-F	0.769 [0.731–0.808]			***z*****= −2.67**, ***p***= 0.008*	***z*****= −3.26**, ***p***= 0.001*
	Model CLIN-COG	0.798 [0.762–0.835]				*z* = −0.67, *p* = 0.504
	Model CLIN-COG-F	0.801 [0.765–0.838]				
		**Threshold**	**Sensitivity**	**Specificity**	**Positive predictive value PPV**	**Negative predictive value NPV**
Sensitivity/Specificity analysis (Method: maximal youden-index)	Model CLIN	0.237	0.691	0.703	0.417	0.881
	Model CLIN-F	0.249	0.663	0.734	0.433	0.878
	Model CLIN-COG	0.299	0.635	0.819	0.518	0.875
	Model CLIN-COG-F	0.290	0.619	0.841	0.544	0.878

Comparison of all less complex models regarding goodness of fit of the underlying multivariate regression analysis and differences between the AUCs of these four models. Columns represent the more complex model; hence, if significance is reached, the model stated in the column has better goodness of fit or greater AUC than the model defined by the name of the row. Analysis of sensitivity and specificity under a maximal Youden-Index can be found below as well. Statistically significant tests results are highlighted in bold type, significant p-values are marked with *, and p-values at the significance level of .05 to 0.1 are marked with (*).

#### Model CLIN

In model FULL, the following four clinical factors were found to be significantly predictive: multimorbidity, renal insufficiency, polypharmacy, and the ASA classification. Three perioperative factors were found to be significantly predictive in model FULL: surgery type, cut-to-suture time, and usage of a CPB. Thus, seven clinical markers were included in the less complex model CLIN, and a multivariate logistic regression was run. The explained variance was 24.2% (*F* = 30.41, *p* < 0.001). Using ROC computation, we determined an AUC of 0.76 (95% CI 0.72, 0.8) for model CLIN.

#### Model CLIN-F

In model CLIN-F, the dichotomized frailty index was included as an addition to the seven clinical markers of model CLIN. This increased the explained variance to 25.6% (*F* = 29.9, *p* < 0.001), and the AUC increased to 0.77 (95% CI 0.73, 0.81), yet this increase did not appear to be significant when compared with model FULL by DeLong's test for two ROC curves (*z* = −1.41, *p* = 0.159). In this model, the association of renal failure was no longer significant.

#### Model CLIN-COG

In model CLIN-COG, cognitive screenings in MoCA, and Digit Span and dementia history were included in addition to the seven clinical markers of model CLIN. This again increased the explained variance to 30.4% (*F* = 30.14, *p* < 0.001). The AUC for this model was 0.8 (95% CI 0.76, 0.84), which proved to be significantly greater than the AUCs of model CLIN (*z* = −3.61, *p* < 0.001) as well as of model CLIN-F (*z* = −2.67, *p* = 0.008) by DeLong's test for two ROC curves. A longer cut-to-suture time was predictive at the level of marginal significance.

#### Model CLIN-COG-F

Finally, 11 predictors were included in a model, clinical and cognitive markers as well as the dichotomized frailty index. In this most complex model CLIN-COG-F, the explained variance increased to 31.4% (*F* = 28.78, *p* < 0.001). The AUC also increased to 0.8 (95% CI 0.77, 0.84), which proved to be significantly higher than the AUC of model CLIN (*z* = −3.5, *p* < 0.001) and that of model CLIN-F (*z* = −3.26, *p* = 0.001), yet not greater than the AUC of model CLIN-COG (*z* = −0.67, *p* = 0.504) by DeLong's test for two ROC curves. Two clinical markers (ASA and multimorbidity), all three cognitive markers—cognitive impairment (MoCA score <23), Digit Span backward (at trend level), and history of dementia—as well as the dichotomized clinical frailty index showed significant ORs.

Multi-collinearity could be ruled out for all factors, as variance-inflation-factors were below 3, besides the one for surgery type (VIF 3.21). For sensitivity analyses, we repeated all analyses subsequently separated for patients receiving orthopedic-general surgery (*n* = 550) or cardiovascular surgery (*n* = 330). In both surgical subgroups the models including cognitive markers lead to an increased explained variance and greater AUCs as compared to the other models. Details and results can be found in the Supplementary Material.

### CHAID Analysis

To better visualize und simplify the multiple regression results, a tree analysis was performed using a CHAID model. Of all the 17 factors and variables included, only four reached significance in the final model and corresponded with a correct classification of 79.3%: first was the ASA classification, second was the grouped cut-to-suture time, additionally, the MoCA score, which was automatically split at 23 (underlining the upper chosen 22/23 cut-off for cognitive impairment (Thomann et al., 2020), and lastly, the clinical frailty score, which was split at three representing the cut-off from being totally autonomously mobile vs. the need for usage of a walking stick. Adding the type of surgery did not alter these results.

**Figure 3:**
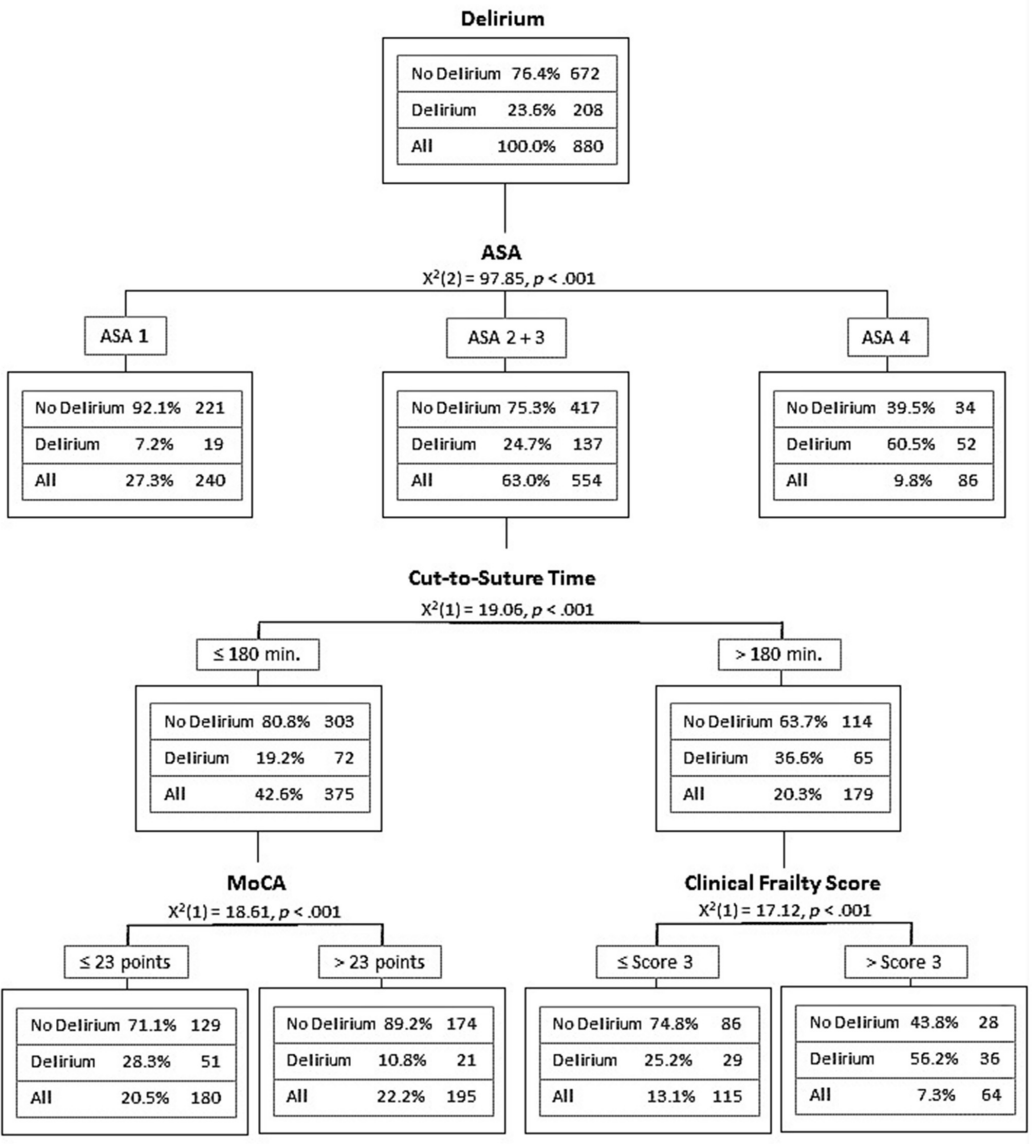
Classification tree showing the CHAID analysis of the final model.

Hence, only four factors were included in the final CHAID analysis: ASA classification (grouped by score I + score II/score III/score IV), C-S-T (grouped by <180 min/>180 min), MoCA score (cut-off < 24), and frailty index CFS (cut-off < 4). Most variance was explained by the ASA score parent node. With an ASA score of I or II, POD risk was 7.9%. The ASA score III group with 554 patients was further split by cut-to-suture time below or beyond 180 min. In case of shorter surgery, patients with a MoCA score > 23 had a POD risk of 10.8%, but with a MoCA score of < 23, a POD risk of 28.3% was calculated. In case of longer surgery, POD risk was elevated to 56% if the clinical frailty scale was score 4 or more. The risk of POD was 25% when non-frail, meaning the CFS was 3 or below. This classification tree was correct for 79.3% of the cases. The predictive performance for no POD was 91%, yet only 42.3% for POD. Figure 3 illustrates the final CHAID analysis.

## Discussion

PAWEL-R was able to estimate stepped delirium risk models for elderly patients undergoing elective surgery in five medical centers. By logistic regression analyses in the whole groups of the participants (lumping various surgical procedures), we determined the following independent main risk factors: high ASA scores, longer expected cut-to-suture time, and preoperative cognitive impairment. Age *per se*, excessive alcohol consumption, active smoking, and visual or hearing impairments were not predictive in the sample.

In the standard clinical risk prediction (Table 4, model CLIN), about 24% of variance (AUC 0.74) were explained in multivariate logistic regression analysis by clinical and perioperative factors. Adding cognitive screening assessments improved the explained variance to about 30% (models CLIN-COG and CLIN-COG-F), while the inclusion of a geriatric assessment (dichotomized clinical frailty index) only increased it to 25.6% (model CLIN-F).

In the Successful Aging after Elective Surgery (SAGES) cohort (Racine et al., 2020), a stepwise logistic regression and machine learning algorithms were used to predict POD in elective surgery of 560 older adults. With a limited data set of 18 items in different machine learning methods, they showed AUCs from 0.53 to 0.57, while in another analysis, adding cognitive performance using the modified Mini-Mental-State 3MS (Teng and Chui, 1987) showed intermediate AUCs (0.53–0.68). With the full data of 71 items, the AUCs in this study were 0.62 up to 0.71. Thus, all prediction models in SAGES were numerically less accurate (Racine et al., 2020) than the shorter models.

The improved predictive accuracy in PAWEL-R might be due to a higher POD incidence in the included cardiovascular surgery group and the usage of the MoCA and Digit Span backward—instead of the MMS or 3MS. The finding of a doubling of delirium risk in the presence of cognitive impairment as evinced by the MoCA scores is comparable with other studies (Racine et al., 2018) and illustrates the importance of comprehensive preoperative cognitive screening. The suggestion of using a lower cut-off score of <24 points could be validated in the CHAID analysis, which showed a risk split at this cut-off.

The phenotype of presurgical cognitive impairment might be important. There is an ongoing debate whether the MoCA (Nasreddine et al., 2005) can be divided into its seven subscores to discriminate different cognitive domains like memory, language, attention, and executive functions. A Memory Index Score (MoCA-MIS) (Julayanont et al., 2014) has been calculated, but the memory index depends on additional questions and scoring from the delayed recall of the five items. As these additional data from delayed recall are not available in the PAWEL sample, cognitive subdomains were not included in the models. All MoCA subscores but naming, however, showed a significant difference between non-delirious and delirious patients. The differences were most pronounced for the memory domain, followed by language and orientation. As the phenotyping of MCI into subtypes like amnestic and non-amnestic MCI is predictive for cognitive decline in dementia (Petersen et al., 2001), we will further analyze MOCA subdomains such as TMT and Digit Span and will differentiate the impact of memory, attention, language, and executive domains on POD risk and potential postoperative cognitive dysfunction (POCD) (Fong et al., 2015b).

Most studies on POD prediction in older adults include multimorbidity or the CCI based on 19 disorders. The acquisition and analysis of the personal medical history is, however, time-consuming. The combination of multimorbidity indices with a frailty score, the easy and short assessment by the seven intuitive levels of the CFS Score (Jones et al., 2005) increased the delirium prediction substantially. In this study, the CFS with a cut-off at 3 vs. 4 (mild frailty in need of a walking aid) added significantly to overall predictive performance for POD.

Regarding different types of surgery, differences between cardiovascular surgery (mostly with CPB) and orthopedic and general surgery were only subtle: The models in orthopedic-general surgery explained less variance and exhibited lower predictive accuracy, possibly because of the lower overall POD incidence in orthopedic and general surgery with overall shorter delirium duration (see Supplementary Material). However, in both groups, the prediction models significantly benefitted from adding cognitive assessments such as MoCA or Digit Span backward. As POD is often an acute reversible brain disorder, brain vulnerability is a central issue and may reflect the finding of a preoperative cognitive impairment as highly predictive of POD. In addition, the results underline the impact of the use of short clinical frailty indices and cognitive impairment as independent risk factors (for cardiovascular surgery, compare Jung et al., 2015).

Overall, cardiovascular surgery is associated with a significantly higher delirium risk than other elective surgeries. Cardiovascular surgery has several procedural implications. In the sample, 87% of the patients received a CPB, which is associated with hypoperfusion of the brain, a higher incidence of delirium (Danielson et al., 2018; Bernardi et al., 2019), and microembolic showers to the cerebral circulation. In the multivariate and tree analysis, however, it was suggested that not the usage of CPB *per se* but the longer duration of cardio-vascular surgery as compared with orthopedic or general surgery, together with the high incidence of severe heart failure, may be the main factor for the higher POD incidence in cardiovascular surgery.

The POD incidence in PAWEL-R was comparable with recent meta-analyses: in vascular surgery, the POD incidence was 23.4% ranging from 4 to 39% (Oldroyd et al., 2017). In elective gastrointestinal surgery, the incidence of POD was between 8 and 55% (Scholz et al., 2016). In the latter analysis, an increased ASA score of III or more was a significant risk factor for POD, while pre-surgical cognitive assessments were not available. The recently reported prevalence of 34% for POD (Itagaki et al., 2020) after cardiac surgery could be confirmed by PAWEL-R. At the same time, older adults who were frail and cognitively impaired showed a significantly increased risk of POD. Another study with 220 cardiac surgery patients and a POD incidence of 52% (Rudolph et al., 2009) identified four independent risk factors for delirium: MMSE impairment, prior stroke or transient ischemic attack, depression, and abnormal albumin. Delirium risk increased more than four-fold from the lowest to highest risk levels. In this study, however, despite the fact that cranial computed tomography **(CCT)** or magnetic resonance imaging (MRI) were not available, predictive accuracy (with an overall AUC of 0.78) was comparable with studies such as neuroimaging. We did not include further comprehensive clinical assessments and biomarkers in the scope of this study.

This risk study initially addressed 18 risk factors. It is not feasible in routine clinical practice to perform such comprehensive risk assessments. Several assessments are quite time consuming and not easily obtained. For example, the TMT-A and TMT-B proved to be time consuming and were obtained from <87% of the sample (Table 1). Likewise, assessments of hyposmia, which was predictive for POD in cardiac surgery (Brown et al., 2015), could just be obtained in <90% of the patients due to temporal constraints and self-reported difficulties to smell. Thus, while the assessments of hyposmia (OR of 1.47) and TMT (TMT-A univariate OR 2.7; TMT-B univariate OR 1.87) might also have independent predictive power, they seem less practicable in routine care.

Precipitating and perioperative risk factors are important to predict POD. The well-known ASA classification and the expected cut-to-suture time have such a predictive power in older adults. Impaired cognitive performance is a major independent risk factor. The MoCA test is such a widely known cognitive assessment and proved to be predictive of delirium, but in itself is a relatively time-consuming tool with a duration of at least 10 min (Nasreddine, 2020). In addition, physical and medical history perioperative factors operationalized in a clinical frailty score together with the type of surgery and its duration exhibited high predictive accuracy. Using <10 risk factors (especially ASA, multimorbidity, expected cut-to-suture time, MoCA, Digit Span backward) we were able to show significant predictive accuracy for delirium in a CHAID analysis and in Model CLIN-COG with an AUC of 0.8. We recommend efficient cognitive items for further evaluation and use in clinical practice.

## Data Availability Statement

The raw data supporting the conclusions of this article will be made available by the authors, without undue reservation.

## Ethics Statement

The studies involving human participants were reviewed and approved by Ethics Commission of the Faculty of Medicine of the Eberhard-Karls University and University Hospital Tübingen with number 233/2017BO1 on October 12, 2017 and by the Ethics Commission of the University of Potsdam with number 38/2017 on December 11, 2017. The study was registered on the German Clinical Trials Register (DRKS-ID: DRKS00012797) in July, 2017. The patients/participants provided their written informed consent to participate in this study. Written informed consent was obtained from the individual(s) for the publication of any potentially identifiable images or data included in this article.

## Author Contributions

GE, CT, and MR designed the study, planned the data collection, performed the statistical analysis, and prepared the manuscript. MC and YK contributed to the data collection and performed the statistical analysis. CA, BM, and MD were involved in designing the study, data collection, and data interpretation. MH, OF, OK, LC, CM, FK, FD, AS, SW, and EM were involved in the data collection and critically revised the manuscript for the final approval of the version to be published. All authors contributed to the article and approved the submitted version.

## Conflict of Interest

The authors declare that the research was conducted in the absence of any commercial or financial relationships that could be construed as a potential conflict of interest.

## Publisher's Note

All claims expressed in this article are solely those of the authors and do not necessarily represent those of their affiliated organizations, or those of the publisher, the editors and the reviewers. Any product that may be evaluated in this article, or claim that may be made by its manufacturer, is not guaranteed or endorsed by the publisher.

## Footnotes

^1^http://id.who.int/icd/entity/897917531.

^2^https://www.destatis.de/DE/Themen/Gesellschaft-Umwelt/Gesundheit/Krankenhaeuser/Publikationen/Downloads-Krankenhaeuser/operationen-prozeduren-5231401197014.html.

^3^https://www.nationale-demenzstrategie.de/fileadmin/nds/pdf/2020-07-01_Nationale_Demenzsstrategie.pdf.

^4^https://www.asahq.org/standards-and-guidelines/asa-physical-status-classification-system.

^5^https://www.r-project.org/.
