# Supplementary material for: Presurgical Screening Improves Risk Prediction for Delirium in Elective Surgery of Older Patients: The PAWEL RISK Study
Source: Front Aging Neurosci. 2021 Jul 27;13:679933. doi: 10.3389/fnagi.2021.679933 (PMC8353451; doi:10.3389/fnagi.2021.679933)
Supplement: Supplementary file 1 [file Data_Sheet_1.PDF]

Supplementary Material

1 Supplement Table 1: Multivariate logistic regression for total sample (excluding dementia)

Results from multivariate logistic regression analysis in order to identify predictors for delirium risk. All data were grouped into the categories stated. Dementia was excluded as a factor, as it was only found in 14 patients of the orthopedic-general group and not at all the cardio-vascular group. The basis for included factors can be found in table 3 in the manuscript. There are only small changes in the OR of the other factors including dementia into regression.

			**Multivariate logistic Regression: Model FULL**	
	N (*n* with delirium); Percentage			750 (177); 23.60 %
	Nagelkerke's Pseudo – R^2			.307
	F			19.08
	p			< .001*
	AUC [95%-CI]			.802 [.764 - .839]
	**Variable**	**Categories**	**OR [95%-CI]**	***p***
	Age	70 - 75 years		
		75 - 80 years	1.08 [0.67 – 1.75]	.745
		80 - 85 years	1.05 [0.58 – 1.89]	.864
		> 85 years	1.36 [0.59 – 3.04]	.466
	ASA	Score I + II		
		Score III	**2.36 [1.25 – 4.61]**	**.009***
		Score IV	**7.34 [3.21 – 17.28]**	**< .001***
	Clinical Frailty Score	Score 1 – 3		
		Score 4 – 8	1.72 [1.10 – 2.71]	**.017***
Poly-pharmacy	Polypharmacy (≤ 5 drugs)	≤ 5 drugs		
		> 5 drugs	1.00 [0.63 – 1.59]	.985
	Polypharmacy (≤ 10 drugs)	≤ 10 drugs		
		> 10 drugs	1.31 [0.71 – 2.37]	.382
	Multimorbidity	≤ 4 diseases		
		5 – 8 diseases	**2.01 [1.18 – 3.50]**	**.010***
		≥ 9 diseases	**4.34 [1.91 – 9.90]**	**< .001***
	Charlson Comorbidity Index	Score 1 + 2		
		Score 3 + 4	0.64 [0.39 – 1.04]	.074
Pre-existing cognitive impairment	MoCA (Nasreddine)	≥ 26 points		
		< 26 points	1.21 [0.72 – 2.05]	.473
	MoCA (< 23)	≥ 23 points		
		< 23 points	**1.82 [1.14 – 2.92]**	**.013***
	Digit Span	≥ 5 points		
		< 5 points	1.42 [0.94 – 2.14]	.099(*)
	Auditory impairment	No ear		
		One ear	1.40 [0.83 – 2.33]	.199
		Both ears	1.37 [0.82 – 2.28]	.219
	Cut-to-Suture-Time	< 90 min.		
		90 – 180 min.	0.57 [0.32 – 1.02]	.059(*)
		> 180 min.	1.80 [0.95 – 3.41]	.071(*)
	Renal Failure	> 45 mmol/ml		
		< 45 mmol/ml	1.49 [0.83 – 2.66]	.175
	Cardio-pulmonal Bypass	No		
		Yes	1.78 [0.91 – 3.59]	.100(*)
	Surgery Type	No		
		Yes	1.21 [0.59 – 2.42]	.590

2 Results for the subsample undergoing orthopedic-general surgery

551 patients received surgery to joints and spine or visceral organs. 16% of these patients suffered from POD within the first week post-surgically. An overview of basic characteristics for this subsample, these data split and compared for patients with and without delirium can be found in table 2, test statistics are given there as well. Stepwise, the minimal multivariate regression models were run upon data from the subsample. Data of 466 patients was included in this analysis, the results can be found in table 3.

Model CLIN explained 13.6% of variance. Out of 6 clinical factors, only the ASA classification and multimorbidity showed significant ORs. An AUC of 0.73 (95%-CI [0.66, 0.79]) was calculated. The addition of the geriatric assessment (CFS) increased the predictive power only marginal to 13.9% in model CLIN-F, and resulted in an AUC of 0.73 (95%-CI [0.68, 0.79]). Therefore, by use of DeLong's test for two ROC curves (z = -0.83, p = .407) no significant increase was shown. In model CLIN-COG, an increase of explained variance to 23.7% was shown, the AUC was 0.77 (95%-CI [0.71, 0.83]) and proved to be significantly increased in comparison to model CLIN (z = -2.38, p = .017) and model CLIN-F (z = -2.18, p = .029). In the most complex model CLIN-COG-F, the explained variance was marginally increased to 23.9% and the AUC again increased to 0.77 (95%-CI [0.72, 0.83]), which was significantly greater than model CLIN (z = -2.46, p = .014) and model CLIN-F (z = -2.38, p = .018), yet not greater than the AUC of model CLIN-COG (z = -0.74, p = .461). Supplementary figure 1 illustrates the computed ROC curves. All statistics regarding the minimal models in comparison can be found in table 4.

2.1 Table 2: Overview of basic characteristics split for patients with and without delirium for orthopedic-general subsample

Overview of basic characteristics and pre- as well as perioperative assessed data of patients without delirium (n = 463) compared with data of patients with delirium (n = 88) undergoing orthopedic or visceral surgery. For continuous data, mean, standard deviation and range are given. If categories were defined or only categorical data was assessed, the categories are defined and the number of patients in each group is given. Test statistics comparing these groups on differences on all available data are stated as well (t-tests were conducted on continuous data, χ^2-tests on categorical; if both types of data were present, both tests were conducted).

	Study subjects without delirium (N = 463)					Study subjects with delirium (N = 88)							
Variable	Mean (+/- SD)	N (available data)	Percentage available data	Categories	N	Mean (+/- SD)	N (available data)	Percentage available data	Categories	N	t	χ^2	p
Age	78.04 (+/- 4.80)	463	100%	70 - 75 years	133	80.15 (+/- 5.53)	88	100%	70 - 75 years	18	-3.24		< .001*
				75 - 80 years	174				75 - 80 years	26			
				80 - 85 years	105				80 - 85 years	25		13.38	.004*
				> 85 years	41				> 85 years	18			
Gender		463	100%	Female	278		88	100%	Female	39		0.42	.519
				Male	185				Male	49			
Education	12.01 (+/- 3.06)	453	97.84%			11.84 (+/- 3.17)	83	94.31%			0.46		.648
ASA	2.53 (+/- 0.58)	457	98.70%	Score I + II	210	2.86 (+/- 0.46)	86	97.73%	Score I + II	14	-4.96		< .001*
				Score III	239				Score III	69		26.49	< .001*
				Score IV	8				Score IV	3			
Clinical Frailty Score	3.79 (+/- 1.36)	457	98.70%	Score 1 – 3	69	4.62 (+/- 1.58)	87	98.86%	Score 1 – 3	4	-4.60		< .001*
				Score 4 – 8	388				Score 4 – 8	83		6.06	.014*
Polypharmacy		463	100%	≤ 5 drugs	221		88	100%	≤ 5 drugs	29		5.93	.015*
				> 5 drugs	242				> 5 drugs	59			
				≤ 10 drugs	403				≤ 10 drugs	65		9.03	.003*
				> 10 drugs	60				> 10 drugs	23			
				≤ 5 drugs	221				≤ 5 drugs	29			
				6 – 10 drugs	182				6 – 10 drugs	36		12.13	.002*
				> 10 drugs	60				> 10 drugs	23			
Multimorbidity	5.79 (+/- 2.54)	463	100%	≤ 4 diseases	155	7.05 (+/- 2.92)	88	100%	≤ 4 diseases	15	-4.15		< .001*
				5 – 8 diseases	272				5 – 8 diseases	61		10.75	.005*
				≥ 9 diseases	36				≥ 9 diseases	12			

Charlson Comorbidity Index	**2.06 (+/- 0.91)**	463	100%	Score 1 + 2	333	**2.34 (+/- 0.92**	88	100%	Score 1 + 2	55	**-2.54**		**.011***
				Score 3 + 4	130				Score 3 + 4	33		2.72	.099(*)
MoCA	**27.07 (+/- 3.81)**	450	97.19%	**≥ 26 points**	**312**	**23.01 (+/- 6.75)**	80	90.91%	**≥ 26 points**	**38**	**5.20**		**< .001***
				**< 26 points**	**138**				**< 26 points**	**42**		**13.48**	**< .001***
				**≥ 23 points**	**403**				**≥ 23 points**	**49**		**41.13**	**< .001***
				**< 23 points**	**47**				**< 23 points**	**31**			
TMT A	**46.56 (+/- 24.07)**	397	85.75%			**73.83 (+/- 54.93)**	65	73.86%			**-3.94**		**< .001***
	**0.26 (+/- 1.47)**			**≥ -1.28 z**	**363**	**-1.25 (+/- 3.20)**			**≥ -1.28 z**	**45**	**3.62**		**.001***
				**< -1.28 z**	**26**				**≥ -1.28 z**	**45**		**21.55**	**< .001***
TMT B	**157.06 (+/- 68.89)**	373	80.56%			**194.21 (+/- 70.92)**	53	60.23%			**-3.58**		**< .001***
	**-0.58 (+/- 1.50)**			**≥ -1.28 z**	**272**	**-1.31 (+/- 1.47)**			**≥ -1.28 z**	**28**	**3.23**		**< .001***
				**< -1.28 z**	**93**				**< -1.28 z**	**22**		**6.63**	**.010***
Digit Span	**5.05 (+/- 1.88)**	424	91.58%	**≥ 5 points**	**259**	**4.34 (+/- 2.04)**	79	89.77%	**≥ 5 points**	**38**	**3.51**		**.001***
				**< 5 points**	**165**				**< 5 points**	**41**		**4.12**	**.042***
Pre-existing Dementia		463	100%	**No**	**462**		88	100%	**No**	**75**		**57.53**	**< .001***
				**Yes**	**1**				**Yes**	**13**			
Alcoholconsumption		463	100%	≤ 3 drinks/day	462		87	98.86%	≤ 3 drinks/day	86		0.13	.720
				> 3 drinks/day	1				> 3 drinks/day	1			
				≤ 3 drinks/week	450				≤ 3 drinks/week	86		0.28	.596
				> 3 drinks/week	13				> 3 drinks/week	1			
Smoking		463	100%	≤ 5 cigarettes	448		88	100%	≤ 5 cigarettes	83		0.66	.417
				> 5 cigarettes	15				> 5 cigarettes	5			
Auditory impairment		453	97.84%	No ear	241		85	96.59%	No ear	35		4.23	.121
				One ear	110				One ear	27			
				Both ears	102				Both ears	23			
Visual impairment		434	93.74%	**No**	**340**		83	94.32%	**No**	**55**		**4.99**	**.026***
				**Yes**	**94**				**Yes**	**28**			
Sensory impairment		433	93.52%	**None**	**34**		82	93.18%	**None**	**12**		**6.70**	**.035***
				**One sense**	**254**				**One sense**	**37**			
				**Both senses**	**145**				**Both senses**	**33**			
Hyposmia	**9.26 (+/-.98)**	378	81.64%	**No**	**273**	**7.79 (+/- 3.02)**	62	70.45%	**No**	**32**	**3.70**		**< .001***

				**Yes**	**105**				**Yes**	**30**		**9.69**	**.002***
Cut-to-Suture-Time	**105.41 (+/- 55.91)**	463	100%	**< 90 min.**	**243**	**127.85 (+/- 94.71)**	88	100%	**< 90 min.**	**47**	**-2.15**		**.034***
				**90 – 180 min.**	**173**				**90 – 180 min.**	**24**		**7.47**	**.024***
				**> 180 min.**	**47**				**> 180 min.**	**17**			
Renal Failure	259.83 (+/- 1588.88)	463	100%	**> 45 mmol/ml**	**396**	228.03 (+/- 1497.22)	88	100%	**> 45 mmol/ml**	**67**	0.17		.862
				**< 45 mmol/ml**	**54**				**< 45 mmol/ml**	**18**		**4.41**	**.036***

2.2 Table 3: Results from multivariate regression analysis for the minimal models in the orthopedic-general subsample

Results from multivariate logistic regression analysis comparing four possible minimal models with high economical practicability for patients undergoing orthopedic or visceral surgery. All data were grouped into the categories stated. To ensure the possibility of statistical comparison of all models, only patients were included, for whom data in all models were available. Variables „cardio-pulmonal bypass“ and „surgery type“ were excluded.

		Model CLIN	Model CLIN-F	Model CLIN-COG	Model CLIN-COG-F
Basic Information	N (N with delirium)	466 (70); 15.02%	466 (70); 15.02%	466 (70); 15.02%	466 (70); 15.02%
	Nagelkerke's Pseudo-R^2	.136	.139	.237	.239
	F	12.04	10.56	14.13	12.96
	p	< .001*	< .001*	< .001*	< .001*
	AUC [95%-CI]	.725 [.663 - .786]	.729 [.679 - .789]	.770 [.713 - .827]	.773 [.717 - .829]

	Variable	Categories	N (N with delirium); Percentage	Model CLIN OR [95%-CI]	*p*	Model CLIN-F OR [95%-CI]	*P*	Model CLIN-COG OR [95%-CI]	*p*	Model CLIN-COG-F OR [95%-CI]	*P*
Clinical	ASA	Score I + II	215 (17); 7.91%								
		Score III	476 (116); 24.37%	**3.70 [1.85 – 7.80]**	**< .001***	**3.55 [1.77 – 7.52]**	**.001***	**3.83 [1.86 – 8.35]**	**< .001***	**3.71 [1.79 – 8.12]**	**< .001***
		Score IV	79 (48); 60.76%	4.93 [0.62 - 27.53]	.086$^{(*)}$	4.62 [0.57 - 26.42]	.103	3.78 [0.27 – 27.48]	.239	3.61 [0.25 – 26.72]	.259
	Multimorbidity	≤ 4 diseases	209 (26); 12.44%								
		5 – 8 diseases	489 (126); 25.77%	**2.15 [1.05 – 4.76]**	**.045***	**2.18 [1.07 - 4.83]**	**.042***	1.97 [0.95 - 4.43]	.082$^{(*)}$	2.00 [0.96 – 4.50]	.077$^{(*)}$
		≥ 9 diseases	72 (29); 40.28%	**3.06 [1.12 – 8.50]**	**.029***	**3.08 [1.12 – 8.57]**	**.029***	2.59 [0.87 – 7.67]	.083$^{(*)}$	0.62 [0.88 – 7.75]	.081$^{(*)}$
	Cut-to-Suture Time	< 90 min.	260 (41); 15.77%								
		90 – 180 min.	256 (38); 14.84%	0.60 [0.31 – 1.11]	.111	0.57 [0.30 - 1.07]	.089$^{(*)}$	0.54 [0.26 – 1.04]	.073	0.52 [0.25 – 1.02]	.062$^{(*)}$
		> 180 min.	254 (102); 40.16%	1.61 [0.74 – 3.37]	.215	1.59 [0.73 – 3.33]	.229	1.84 [0.83 - 3.91]	.121	1.82 [0.82 – 3.87]	.128
	Renal Failure	> 45 mmol/ml	671 (148); 22.06%								
		< 45 mmol/ml	99 (33); 33.33%	1.09 [0.52 – 2.16]	.816	1.04 [0.50 - 2.09]	.908	0.85 [0.37 – 1.80]	.676	0.82 [0.35 – 1.75]	.616
	Polypharmacy	≤ 5 drugs	339 (60); 17.70%								
		6 – 10 drugs	337 (88); 26.11%	0.78 [0.41 – 1.50]	.464	0.75 [0.38 - 1.44]	.383	0.69 [0.34 – 1.37]	.293	0.66 [0.33 – 1.33]	.250
		> 10 drugs	94 (33); 35.11%	1.19 [0.55 – 2.58]	.653	1.07 [0.47 - 2.38]	.877	0.98 [0.42 - 2.24]	.960	0.89 [0.37 – 2.10]	.797
Cognition	MoCA	≥ 23 points	516 (100); 19.38%								
		< 23 points	254 (81); 31.89%					**2.12 [1.16 – 3.87]**	**.014***	**2.07 [1.13 – 3.80]**	**.018***

	Digit Span	≥ 5 points	459 (86); 18.74%								
		< 5 points	311 (95); 30.55%					1.08 [0.60 – 1.95]	.793	1.08 [0.59 – 1.95]	.803
	Pre-existing Dementia	No	760 (172); 22.63%								
		Yes	10 (9); 90.00%					**31.91 [5.37 – 614.12]**	**.002***	**32.40 [5.44 – 623.76]**	**.002***
Frailty	Clinical Frailty Score	Score 1 – 3	440 (89); 20.23%								
		Score 4 – 8	330 (92); 27.89%			1.35 [0.74 - 2.48]	.327			1.29 [0.69 - 2.44]	.432

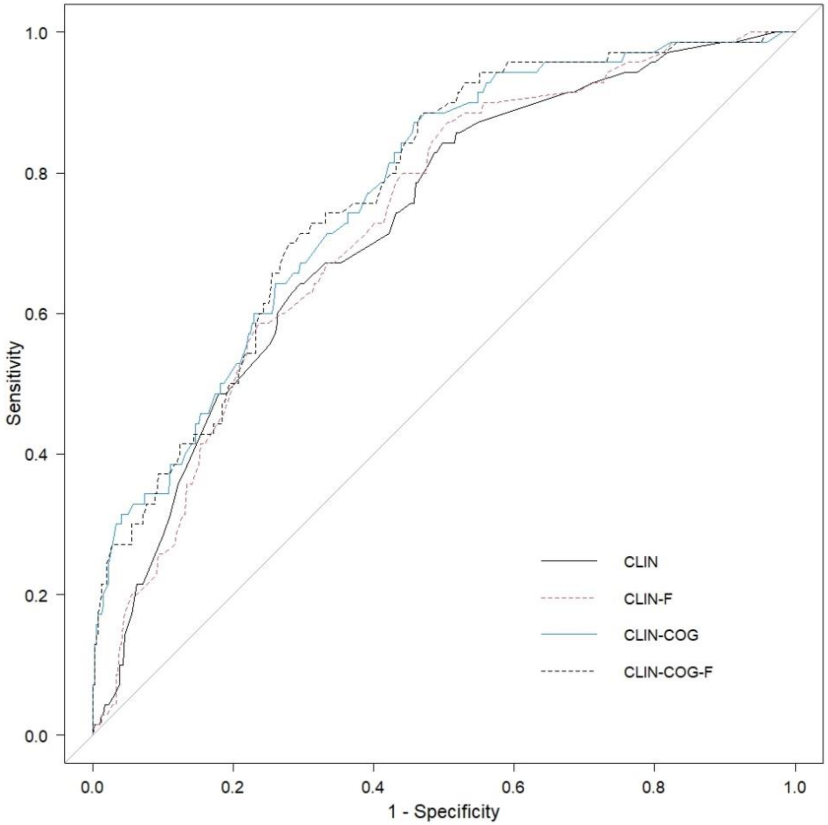

Supplementary Figure 1. ROC-curves of minimal models for subsample undergoing orthopedic-general surgery.

2.3 Table 4: Comparison of 4 models under several aspects for the orthopedic-general subsample

Comparison of all 4 models regarding the goodness of fit of the underlying multivariate regression analysis and differences between the AUCs of these four models analyzing patients undergoing orthopedic or visceral surgery. Columns represent the more complex model, hence, if significance is reached, the model stated in the column has a better goodness of fit or greater AUC than the model defined by the row's name. Analysis of sensitivity and specificity under the aspect of a maximal Youden-Index can be found as well.

		**Pseudo – R^2**	**Model CLIN**	**Model CLIN-F**	**Model CLIN-COG**	**Model CLIN-COG-F**
Likelihood ratio tests	Model CLIN	.136		χ^2 (1) = 0.962, *p* = .327*	**χ^2 (3) = 30.12, *p* < .001***	**χ^2 (4) = 30.74, *p* < .001***
	Model CLIN-F	.139			**χ^2 (2) = 29.16, *p* < .001***	**χ^2 (3) = 29.78, *p* < .001***
	Model CLIN-COG	.237				χ^2 (1) = 0.62, *p* = .431
	Model CLIN-COG-F	.239				
		**AUC [95%-CI]**				
DeLong's tests for two ROC-curves in a correlated population	Model CLIN	.725 [.663 - .786]		*z* = -0.83, *p* = .407	***z* = -2.38, *p* = .017***	***z* = -2.46, *p* = .014***
	Model CLIN-F	.729 [.679 - .789]			***z* = -2.18, *p* = .029***	***z* = -2.38, *p* = .018***
	Model CLIN-COG	.770 [.713 - .827]				*z* = -0.74, *p* = .461
	Model CLIN-COG-F	.773 [.717 - .829]				
		**Threshold**	**Sensitivity**	**Specificity**	**Positive Predictive Value PPV**	**Negative Predictive Value NPV**
Sensitivity / Specificity analysis *(Maximal Youden-Index)*	Model CLIN	.187	.643	.705	.278	.918
	Model CLIN-F	.108	.871	.495	.236	.949
	Model CLIN-COG	.106	.871	.543	.252	.960
	Model CLIN-COG-F	.163	.700	.720	.306	.931

3 Results for subsample undergoing cardio-vascular surgery

329 patients received surgery at the heart or big vessels. 87% of anesthesia were assisted by a CPB. 36% of these suffered from POD within the first week post-surgically. An overview of basic characteristics for this subsample, these data split and compared for patients with and without delirium can be found in table 5, test statistics are given there as well. Stepwise, the minimal multivariate regression models were run upon data from the subsample. Data of 329 patients was included in this analysis, the results can be found in table 6.

Model CLIN explained 26.5% of variance. 3 out of 7 included clinical factors explained variance significantly: Multimorbidity, kidney insufficiency and cut-suture-time, but the ASA classification and usage of CPB were not predictive. The AUC of model CLIN was 0.76 (95%-CI [0.70, 0.83]). In model CLIN-F, the explained variance increased to 29.7%, an AUC of 0.78 (95%-CI [0.73-0.83]) was calculated, which was marginally significantly higher than the AUC of model CLIN (z = -1.74, p = .082) under DeLong's test for two ROC curves. In model CLIN-COG the explained variance raised to 30.0% with an AUC of 0.79 (95%-CI [0.74, 0.84]), which was greater than model CLIN (z = -2.07, p = .038) yet not higher than model CLIN-F (z = -0.60, p = .552). In this model, renal insufficiency with an OR of 3.31 for renal insufficiency as well as the Digit Span backwards with an OR of 2.06 were significant predictors. In model CLIN-COG-F, the explained variance was highest with 33.3% and the AUC was 0.80 (95%-CI [0.78, 0.85]). This again was higher than model CLIN (z = -2.37, p = .018), marginally significant greater than model CLIN-F (z = -1.68, p = .093), yet not higher than model CLIN-COG (z = -1.28, p = .202). Supplementary figure 2 illustrates the computed ROC curves. All statistics regarding the 4 models in comparison can be found in table 7.

3.1 Table 5: Overview of basic characteristics split for patients with and without delirium for cardio-vascular subsample

Overview of basic characteristics and pre- as well as perioperative assessed data of patients without delirium (*n* = 209) compared with data of patients with delirium (*n* = 120) undergoing cardio-vascular surgery. For continuous data, mean, standard deviation and range are given. If categories were defined or only categorical data was assessed, the categories are defined and the number of patients in each group is given. Test statistics comparing these groups on differences on all available data are stated as well (*t*-tests were conducted on continuous data, $\chi2$-tests on categorical; if both types of data were present, both tests were conducted).

	**Study subjects without delirium (*N* = 209)**					**Study subjects with delirium (*N* = 120)**							
**Variable**	**Mean (+/- SD)**	***N* (available data)**	**Percentage available data**	**Categories**	***N***	**Mean (+/- SD)**	***N* (available data)**	**Percentage available data**	**Categories**	***N***	***t***	**X^2**	***p***
Age	76.85 (+/- 4.55)	209	100%	70 - 75 years	81	77.05 (+/- 4.58)	120	100%	70 - 75 years	40	-0.39		.699
				75 - 80 years	79				75 - 80 years	53			
				80 - 85 years	37				80 - 85 years	21		1.75	.627
				> 85 years	12				> 85 years	5			
Gender		209	100%	Female	66		120	100%	Female	41		0.13	.719
				Male	143				Male	79			
Education	12.78 (+/- 2.85)	208	99.52%			12.37 (+/- 2.59)	119	99.17%			1.30		.193
ASA	**3.06 (+/- 0.45)**	208	99.52%	**Score I + II**	**11**	**3.37 (+/- 0.56)**	120	100%	**Score I + II**	**5**	**-5.35**		**< .001***
				**Score III**	**171**				**Score III**	**66**		**34.71**	**< .001***
				**Score IV**	**26**				**Score IV**	**49**			
Clinical Frailty Score	**2.88 (+/- 0.89)**	208	99.52%	**Score 1 – 3**	**71**	**3.36 (+/- 1.15)**	119	99.17%	**Score 1 – 3**	**24**	**-3.98**		**< .001***
				**Score 4 – 8**	**137**				**Score 4 – 8**	**95**		**6.50**	**.011***
Polypharmacy		209	100%	**≤ 5 drugs**	**90**		120	100%	**≤ 5 drugs**	**37**		**4.31**	**.038***
				**> 5 drugs**	**119**				**> 5 drugs**	**83**			
				**≤ 10 drugs**	**199**				**≤ 10 drugs**	**104**		**6.52**	**.011***
				**> 10 drugs**	**10**				**> 10 drugs**	**16**			
				**≤ 5 drugs**	**90**				**≤ 5 drugs**	**37**			
				**6 – 10 drugs**	**109**				**6 – 10 drugs**	**67**		**10.20**	**.006***
				**> 10 drugs**	**10**				**> 10 drugs**	**16**			
Multimorbidity	**6.31 (+/- 2.35)**	209	100%	**≤ 4 diseases**	**50**	**6.92 (+/- 2.50)**	120	100%	**≤ 4 diseases**	**16**	**-2.22**		**.027***

				**5 – 8 diseases**	**146**				**5 – 8 diseases**	**84**		**12.56**	**.002***
				**≥ 9 diseases**	**13**				**≥ 9 diseases**	**20**			
Charlson Comorbidity Index	2.22 (+/- 0.84)	209	100%	Score 1 + 2	140	2.31 (+/- 0.90)	120	100%	Score 1 + 2	78	-0.94		.347
				Score 3 + 4	69				Score 3 + 4	42		0.06	.806
MoCA	**27.98 (+/- 3.22)**	208	99.52%	**≥ 26 points**	**170**	**26.70 (+/- 3.40)**	117	97.50%	**≥ 26 points**	**80**	**3.32**		**.001***
				**< 26 points**	**38**				**< 26 points**	**37**		**6.79**	**.009***
				≥ 23 points	197				≥ 23 points	103		3.81	.051(*)
				< 23 points	11				< 23 points	14			
TMT A	44.91 (+/- 26.37)	203	97.60%			46.56 (+/- 18.34)	112	93.33%			-0.65		.514
	0.38 (+/- 1.81)			≥ -1.28 z	190	0.28 (+/- 1.12)			≥ -1.28 z	101	0.62		.533
				< -1.28 z	12				≥ -1.28 z	11		1.08	.299
TMT B	**144.72 (+/- 66.04)**	197	94.26%			**179.07 (+/- 75.34)**	109	90.83%			**-3.99**		**< .001***
	**-0.25 (+/- 1.30)**			**≥ -1.28 z**	**159**	**-0.89 (+/- 1.66)**			**≥ -1.28 z**	**74**	**3.69**		**< .001***
				**< -1.28 z**	**37**				**< -1.28 z**	**35**		**6.09**	**.014***
Digit Span	**5.69 (+/- 2.39)**	200	95.69%	**≥ 5 points**	**138**	**4.95 (+/- 2.31)**	116	96.67%	**≥ 5 points**	**53**	**3.01**		**.003***
				**< 5 points**	**62**				**< 5 points**	**63**		**15.72**	**< .001***
Pre-existing Dementia		209	100%	**No**	**209**		120	100%	**No**	**120**			
				**Yes**	**0**				**Yes**	**0**			
Alcoholconsumption		207	99.04%	≤ 3 drinks/day	206		120	100%	≤ 3 drinks/day	120		< 0.01	1
				> 3 drinks/day	1				> 3 drinks/day	0			
				≤ 3 drinks/week	201				≤ 3 drinks/week	115		0.09	.768
				> 3 drinks/week	6				> 3 drinks/week	5			
Smoking		209	100%	≤ 5 cigarettes	196		120	100%	≤ 5 cigarettes	113		< 0.01	1
				> 5 cigarettes	13				> 5 cigarettes	7			
Auditory impairment		208	99.52%	No ear	140		119	99.17%	No ear	71		3.01	.222
				One ear	29				One ear	16			
				Both ears	39				Both ears	32			
Visual impairment		203	97.13%	No	164		119	99.17%	No	99		0.15	.697
				Yes	29				Yes	20			
Sensory impairment		202	96.65%	None	28		118	98.33%	None	12		2.52	.284

				One sense	121				One sense	66			
				Both senses	53				Both senses	40			
Hyposmia	9.09 (+/- 2.47)	182	87.08%	No	124	8.86 (+/- 2.40)	106	88.33%	No	70	0.77		.440
				Yes	58				Yes	36		0.06	.814
**Cut-to-Suture-Time**				**< 90 min.**	**13**				**< 90 min.**	**2**	**-5.36**		**< .001***
	**194.06 (+/- 71.83)**	209	100%	**90 – 180 min.**	**79**	**239.13 (+/-73.95)**	119	99.17%	**90 – 180 min.**	**20**		**22.06**	**< .001***
				**> 180 min.**	**117**				**> 180 min.**	**97**			
Renal Failure	144.41 (+/- 1191.00)	209	100%	> 45 mmol/ml	189	1.073 (+/- 0.36)	120	100%	> 45 mmol/ml	100	1.32		.189
				< 45 mmol/ml	17				< 45 mmol/ml	19		3.81	.051(*)
Cardio-pulmonal Bypass				**No**	**67**				**No**	**25**			
		209	100%	**Yes**	**142**		120	100%	**Yes**	**95**		**4.23**	**.040***

3.2 Table 6: Results from multivariate regression analysis for the minimal models in the cardio-vascular subsample

Results from multivariate logistic regression analysis comparing four possible minimal models with high economical practicability for patients undergoing cardio-vascular surgery. All data were grouped into the categories stated. To ensure the possibility of statistical comparison of all models, only patients were included, for whom data in all models were available. Variables „pre-existing dementia“ and „surgery type“ were excluded.

				Model CLIN		Model CLIN-F		Model CLIN-COG		Model CLIN-COG-F	
Basic Information	N (N with delirium)			307 (111); 36.16%		307 (111); 36.16%		307 (111); 36.16%		307 (111); 36.16%	
	Nagelkerke's Pseudo-R^2			.265		.298		.311		.334	
	F			15.40		15.81		14.90		14.84	
	p			< .001*		< .001*		< .001*		< .001*	
	AUC [95%-CI]			.759 [.702 - .826]		.781 [.727 - .834]		.790 [.737 - .843]		.801 [.479 - .853]	
	**Variable**	**Categories**	**N (N with delirium); Percentage**	**OR [95%-CI]**	***p***	**OR [95%-CI]**	***P***	**OR [95%-CI]**	***p***	**OR [95%-CI]**	***P***
Clinical	ASA	Score I + II	15 (4); 28.57%								
		Score III	221 (61); 27.60%	0.65 [1.18 – 2.72]	.529	0.61 [1.16 – 2.67]	.482	0.76 [0.20 – 3.29]	.690	0.69 [1.18 – 3.14]	.617
		Score IV	71 (46); 64.79%	2.70 [0.71 – 11.84]	.159	2.45 [0.61 – 11.22]	.219	2.82 [0.71 – 12.91]	.153	2.55 [0.61 – 12.11]	.213
	Multimorbidity	≤ 4 diseases	65 (16); 24.61%								
		5 – 8 diseases	211 (77); 36.49%	1.52 [0.77 – 3.10]	.228	1.45 [0.73 – 2.96]	.296	1.44 [0.71 – 3.05]	.323	1.37 [0.67 – 2.89]	.432
		≥ 9 diseases	31 (18); 58.06%	**3.99 [1.43 – 11.53]**	**.009***	**2.95 [1.01 – 8.79]**	**.048***	3.76 [1.31 – 11.11]	.014(*)	2.86 [0.97 – 8.62]	.063(*)
	Cut-to-Suture Time	< 90 min.	13 (2); 15.38%								
		90 – 180 min.	94 (20); 21.28%	2.89 [0.54 – 23.39]	.252	2.81 [0.48 – 24.05]	.286	3.13 [0.55 – 26.41]	.233	3.06 [0.51 – 26.84]	.257
		> 180 min.	200 (89); 44.50%	**7.38 [1.37 – 60.30]**	**.032***	**7.49 [1.27 – 65.47]**	**.039***	8.20 [1.43 – 69.95]	.029(*)	**8.23 [1.35 – 73.61]**	**.034***
	Renal Failure	> 45 mmol/ml	271 (92); 33.95%								
		< 45 mmol/ml	36 (19); 52.78%	**2.85 [1.22 – 6.92]**	**.017***	**2.69 [1.12 – 6.6/]**	**.029***	**3.32 [1.38 – 8.35]**	**.008***	**3.08 [1.26 – 7.81]**	**.015***
	Polypharmacy	≤ 5 drugs	122 (35); 28.69%								
		6 – 10 drugs	160 (61); 38.13%	1.59 [0.90 – 2.85]	.114	1.40 [0.78 – 2.5%]	.265	1.33[0.73 – 2.44]	.345	1.21 [0.66 – 2.25]	.515
		> 10 drugs	25 (15); 60.00%	2.64 [0.90 – 7.94]	.078 (*)	2.04 [0.67 – 6.3§]	.211	2.02 [0.67 – 6.16]	.211	1.64 [0.53 – 5.12]	.387
	Cardio-pulmonal Bypass	No	80 (20); 25.00%								

		Yes	227 (91); 40.09%	1.44 [0.73 – 2.942]	.304	1.68 [0.84 – 3.46]	.150	1.62 [0.81 – 3.35]	.182	1.87 [0.92 – 3.93]	.087$^{(*)}$
Cognition	MoCA	≥ 23 points	221 (69); 31.22%					1.75 [0.94 – 3.26]	.078$^{(*)}$		
		< 23 points	86 (42); 48.84%							1.50 [0.79 – 2.85]	.219
	Digit Span	≥ 5 points	185 (50); 27.03%					**2.07 [1.17 – 3.69]**	**.013***		
		< 5 points	122 (61); 50.00%					1.75 [0.94 – 3.26]	.078$^{(*)}$	**2.10 [1.17 – 3.79]**	**.013***
Frailty	Clinical Frailty Score	Score 1 – 3	221 (64); 28.96%								
		Score 4 – 8	86 (47); 54.65%			**2.58 [1.39 – 4.85]**	**.003***			**2.33 [1.23 – 4.46]**	**.010***

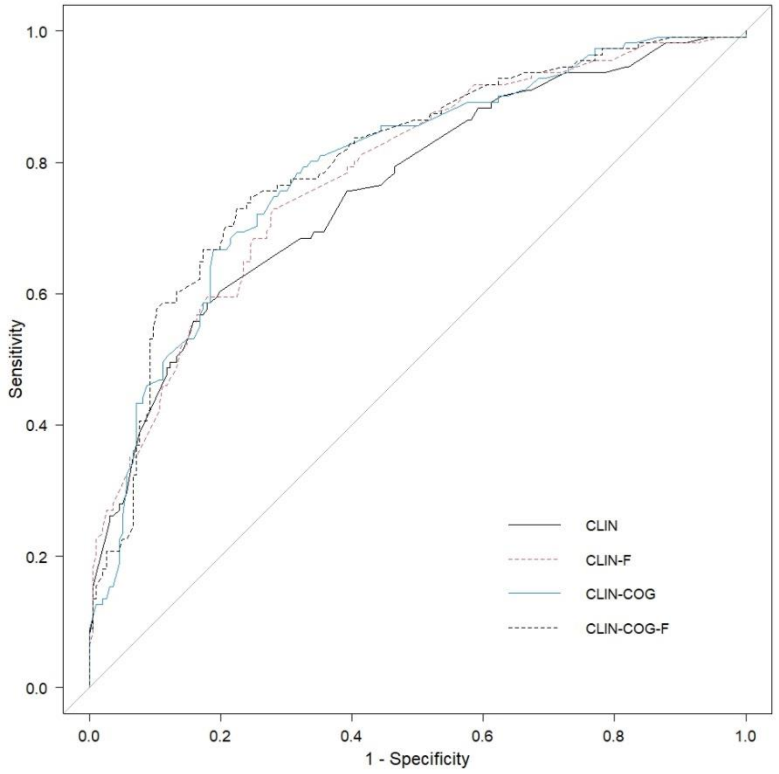

Supplementary Figure 2. ROC-curves of 4 models for subsample undergoing cardio-vascular surgery.

3.3 Table 7: Comparison of all minimal models under several aspects for the cardio-vascular subsample

Comparison of all minimal models regarding the goodness of fit of the underlying multivariate regression analysis and differences between the AUCs of these four models analyzing patients undergoing cardio-vascular surgery. Columns represent the more complex model, hence, if significance is reached, the model stated in the column has a better goodness of fit or greater AUC than the model defined by the row's name. Analysis of sensitivity and specificity under the aspect of a maximal Youden-Index.

		**Pseudo – R^2**	**Model CLIN**	**Model CLIN-F**	**Model CLIN-COG**	**Model CLIN-COG-F**
Likelihood ratio tests	Model CLIN	.265		**χ^2 (1) = 9.06, p = .003***	**χ^2 (2) = 12.82, p = .002***	**χ^2 (3) = 19.58, p < .001***
	Model CLIN-F	.297			χ^2 (1) = 3.76, p = .053(*)	**χ^2 (2) = 10.52, p = .005***
	Model CLIN-COG	.300				**χ^2 (1) = 6.76, p = .009***
	Model CLIN-COG-F	.333				
		**AUC [95%-CI]**				
DeLong's tests for two ROC-curves in a correlated population	Model CLIN	.759 [.702 - .826]		z = - 1.74, p = .082(*)	**z = - 2.07, p = .038***	**z = - 2.37, p = .018***
	Model CLIN-F	.781 [.727 - .834]			z = - 0.60, p = .552	z = - 1.68, p = .093(*)
	Model CLIN-COG	.790 [.737 - .843]				z = - 1.28, p = .202
	Model CLIN-COG-F	.801 [.779 - .853]				
		**Threshold**	**Sensitivity**	**Specificity**	**Positive Predictive Value PPV**	**Negative Predictive Value NPV**
Sensitivity / Specificity analysis *(Maximal Youden-Index)*	Model CLIN	.387	.586	.821	.650	.778
	Model CLIN-F	.312	.730	.719	.591	.824
	Model CLIN-COG	.420	.667	.811	.667	.811
	Model CLIN-COG-F	.381	.730	.776	.655	.809
