# Supplementary material for: Presurgical Screening Improves Risk Prediction for Delirium in Elective Surgery of Older Patients: The PAWEL RISK Study
Source: Front Aging Neurosci. 2021 Jul 27;13:679933. doi: 10.3389/fnagi.2021.679933 (PMC8353451; doi:10.3389/fnagi.2021.679933)
Supplement: Supplementary file 2 [file Data_Sheet_2.PDF]

Supplement S2: Table 1 ASA Physical Status Classification

https://www.asahq.org/standards-and-guidelines/asa-physical-status-classification-system
(Approved by the ASA House of Delegates on October 15, 2014): The ASA Physical Status Classification System alone does not predict the perioperative risks, but used with other factors (eg, type of surgery, frailty, level of deconditioning), it can be helpful in predicting perioperative risks. The definitions and examples of adult patients shown in the table below are guidelines for the clinician. Assigning a Physical Status classification level is a clinical decision based on multiple factors. While the Physical Status classification may initially be determined at various times during the preoperative assessment of the patient, the final assignment of Physical Status classification is made on the day of anesthesia care by the anesthesiologist after evaluating the patient. Current definitions and ASA-Approved examples for level ASA I to IV, which are relevant or elective surgery. ASA V is only for emergency surgery.

**ASA I**	A normal healthy patient	Healthy, non-smoking, no or minimal alcohol use
**ASA II**	A patient with mild systemic disease	Mild diseases only without substantive functional limitations. Current smoker, social alcohol drinker, pregnancy, obesity (30<BMI<40), well-controlled DM/HTN, mild lung disease
**ASA III**	A patient with severe systemic disease	Substantive functional limitations; One or more moderate to severe diseases. Poorly controlled DM or HTN, COPD, morbid obesity (BMI ≥40), active hepatitis, alcohol dependence or abuse, implanted pacemaker, moderate reduction of ejection fraction, ESRD undergoing regularly scheduled dialysis, history (>3 months) of MI, CVA, TIA, or CAD/stents.
**ASA IV**	A patient with severe systemic disease that is a constant threat to life	Recent (<3 months) MI, CVA, TIA or CAD/stents, ongoing cardiac ischemia or severe valve dysfunction, severe reduction of ejection fraction, shock, sepsis, DIC, ARD or ESRD not undergoing regularly scheduled dialysis

ARD: acute respiratory distress, BMI: body mass index, CAD: coronary artery disease, COPD: chronic obstructive lung disease, CVA: cerebrovascular accident, DIC: disseminated intravascular coagulation, DM: diabetes mellitus, ESRD: end-stage renal disease, HTN: hypertension, MI: myocardial infarction, TIA transient ischemic attack

Supplement 2, Table 2 Overview of the individual seven MoCA categories (Nasreddine et al. 2005) of patients without delirium (*n* = 672) compared with data of patients with delirium (*n* = 208). Median and distribution across categories are given. Test statistics comparing these groups for differences on all available data are stated as well (Mann-Whitney-U-tests were conducted as the data is not normally distributed).

	Study subjects without delirium (*n* = 672)					Study subjects with delirium (*n* = 208)							
**Variable**	**Median**	***N* (available data)**	**Percentage available data**	**Categories**	***N***	**Median**	***N* (available data)**	**Percentage available data**	**Categories**	***N***	***W***	**χ^2**	***p***
Visuospatial / Executive	**4**	659	98.07%	0 points	**10**	**3**	198	95.19%	0 points	**13**	**72135**		**.020***
				1 point	**35**				1 point	**19**			
				2 points	**104**				2 points	**34**			
				3 points	**173**				3 points	**45**			
				4 points	**197**				4 points	**51**			
				5 points	**133**				5 points	**36**			
Naming	3	666	99.12%	0 points	2	3	205	98.56%	0 points	2	70768		.083
				1 point	1				1 point	5			
				2 points	42				2 points	14			
				3 points	621				3 points	184			
Attention	**6**	672	100%	0 points	**6**	**5**	208	100%	0 points	**5**	**78136**		**.005***
				1 point	**5**				1 point	**4**			
				2 points	**6**				2 points	**5**			
				3 points	**26**				3 points	**15**			
				4 points	**76**				4 points	**27**			
				5 points	**312**				5 points	**63**			
				6 points	**340**				6 points	**89**			
Language	**2**	672	100%	0 points	**56**	**2**	208	100%	0 points	**36**	**83623**		**< .001***
				1 point	**174**				1 point	**63**			
				2 points	**273**				2 points	**81**			
				3 points	**169**				3 points	**28**			
Abstraction	**2**	666	99.12%	0 points	**67**	**2**	205	98.56%	0 points	**22**	**75931**		**.005***
				1 point	**170**				1 point	**76**			
				2 points	**429**				2 points	**107**			
Delayed Recall	**3**	666	99.12%	0 points	**129**	**1**	205	98.56%	0 points	**73**	**86273**		**< .001***
				1 point	**69**				1 point	**31**			
				2 points	**129**				2 points	**36**			
				3 points	**139**				3 points	**32**			
				4 points	**113**				4 points	**22**			
				5 points	**87**				5 points	**11**			
Orientation	**6**	666	99.12%	0 points	**0**	**6**	205	98.56%	0 points	**0**	**79454**		**< .001***
				1 point	**0**				1 point	**0**			
				2 points	**0**				2 points	**7**			
				3 points	**1**				3 points	**6**			
				4 points	**4**				4 points	**6**			
				5 points	**42**				5 points	**28**			
				6 points	**619**				6 points	**158**			
